# Mitochondrial dynamics: A promising tool for personalized TNBC management

**DOI:** 10.1016/j.gendis.2026.102157

**Published:** 2026-03-21

**Authors:** Harshit Mishra, Anshu Yadav, Veena B. Kushwaha, Manish Pratap Singh

**Affiliations:** Department of Zoology, Deen Dayal Upadhyaya Gorakhpur University, Gorakhpur, Uttar Pradesh, 273009, India

**Keywords:** Mitochondrial dynamics, Mitochondrial energetics, Personalized medicine, Targeted therapy, TNBC

## Abstract

Triple-negative breast cancer (TNBC) is an aggressive and highly metastatic form of breast cancer and is associated with poor prognosis due to the lack of targeted therapies. Mitochondrial dysfunction is a crucial factor contributing to tumor growth and chemoresistance in TNBC. Dysregulation in mitochondrial dynamics leads to disruption of the normal process of oxidative phosphorylation, elevated reactive oxygen species generation, and resistance to apoptosis, contributing to TNBC aggressiveness. Moreover, alterations in mitochondrial energetics, including elevated glycolysis and glutamine addiction, provide metabolic advantages for TNBC growth and survival. Emerging therapeutic strategies targeting mitochondrial vulnerabilities have shown potential for TNBC management. Inhibitors targeting mitochondrial dynamics, including Mdivi-1, dynasore, and cepharanthine, act by restoring mitochondrial homeostasis and impairing excessive fission to stimulate apoptosis. Mitochondrial energetics inhibitors, such as 2DG, 3BP, clotrimazole, and etomoxir, disrupt mitochondrial metabolic processes and reduce tumor growth in TNBC. This review highlights the latest advancements in mitochondrial dynamics and energetics in TNBC and explores the molecular mechanisms underlying their dysregulation. It also explores the therapeutic potential of targeting mitochondrial function for personalized strategies leading to improved clinical management of TNBC.

## Introduction

Breast cancer (BC) is the most prevalent malignancy and the leading cause of cancer-related deaths among women globally. According to the GLOBOCAN data, BC ranks fifth worldwide in deaths related to cancer, with 685,000 deaths (6.9%) and an estimated 2.3 million newly diagnosed cases from 185 countries, representing 11.7% of all cancer cases globally.^1^ It is a highly malignant tumor divided into four subtypes based on immunohistochemical expression of hormone receptors: estrogen receptor-positive (ER^+^ BC), progesterone receptor-positive (PR^+^ BC), human epidermal growth factor receptor-positive (HER2^+^ BC), and triple-negative breast cancer (TNBC).^2^ Lack of expression of human epidermal growth factor receptor 2 (HER2), progesterone receptor (PR), and estrogen receptor (ER) is the defining characteristic of TNBC, which is responsible for about 15%–20% of BC cases worldwide.^3^ TNBC, with considerable differences in genomic and transcriptomic profiles, is considered a heterogeneous disease.^4^ TNBC is peculiarly linked to higher-grade tumors, early age onset, increased risk of distant metastasis, shorter overall survival, different metabolic phenotypes, and more pronounced dysregulation of cellular metabolism in comparison to other BC subtypes.^5^^,^^6^ TNBC also exhibits common patterns of metabolic adaptations, such as down-regulation of fatty acid degradation and up-regulation of purine and pyrimidine metabolism. Oxidative phosphorylation (OXPHOS) is also up-regulated in TNBC tumor samples compared with normal tissues, while pathways linked to carbohydrate and lipid metabolism exhibit the most variations in TNBC.^5^ *De novo* metastatic disease affects only 5% of TNBC patients, and most of them relapse after receiving treatment.^7^^,^^8^ Usually, TNBC spreads to the liver, lungs, and brain.^9^ Lehmann and Pietenpol have classified it into six subtypes based on their gene expression profiling: immunomodulatory (IM), mesenchymal (M), mesenchymal stem cell-like (MSL), basal-like 1 (BL1), basal-like 2 (BL2), and luminal androgen receptor (LAR).^10^ While Liu et al analyzed 165 tumor samples of TNBC and based on expression analysis of long non-coding RNA (lncRNA) and mRNA, they grouped them into four categories: basal-like immune-suppressed (BLIS), immunomodulatory (IM), mesenchymal (MES), and luminal androgen receptor (LAR).^11^ BLIS is enriched in molecules and pathways like lncRNA TCONS_00000027, mitosis, replication, and DNA repair. The IM subtype includes immune function-related genes CD1C, CXCL13, CXCL10, CCR2, CXCL10, and CCL5, along with lncRNA ENST00000443397. On the other hand, the ENST00000447908 lncRNA and hormone regulation signaling were more abundant in the LAR subtype. The MES subtype includes pathways and genes supporting epithelial–mesenchymal transition, such as lncRNA NR_003221.^3^^,^^11^ Due to the absence of targeted therapies, chemotherapy remains the most crucial strategy to treat TNBC. However, frequent development of chemoresistance results in tumor recurrence and poor patient outcomes.^12^

Mitochondria, the cell’s energy hubs, generate the major part of cellular ATP through OXPHOS and also play crucial roles in regulating cell survival, apoptosis, calcium homeostasis, and redox balance.^13^ The literature review suggests that mitochondrial dysfunction is a hallmark of cancer that contributes to initiation, progression, and chemoresistance in TNBC.^14^ Disruptions in the mitochondrial function, particularly in energy metabolism^15^ and dynamics,^16^ are linked with the development and progression of TNBC.

Mitochondrial dynamics is described as the constant remodeling of mitochondria through fusion and fission, processes critical to maintain mitochondrial functionality and enabling adaptation to cellular stress.^17^ Dynamin-related protein 1 (DRP1) drives the mitochondrial fission, while the fusion of mitochondria is facilitated by the proteins, mitofusin 1 (MFN1) and mitofusin 2 (MFN2), the mitofusins, and optic atrophy 1 (OPA1).^16^ Imbalance in the machinery of fission and fusion results in mitochondrial fragmentation, impaired OXPHOS, elevated production of reactive oxygen species (ROS), and apoptosis resistance.^18^ These mitochondrial abnormalities are frequently observed in TNBC and correlate with poor clinical outcomes.^19^

Metabolic reprogramming is an emerging hallmark of cancer,^20^ and TNBC exhibits a distinct metabolic phenotype characterized by elevated glycolysis and glutamine addiction.^21^ Cancer cells possess a distinct characteristic of the Warburg effect or aerobic glycolysis, in which they prefer the conversion of glucose to lactate, even when oxygen is present.^22^ This metabolic shift provides cancer cells with biosynthetic precursors for rapid proliferation and confers resistance to apoptosis.^23^ Glutamine, another crucial nutrient for TNBC growth and survival, serves as a nitrogen and carbon source for biosynthesis and energy production.^21^ Targeting the metabolic vulnerabilities of TNBC has been identified as a promising treatment approach.^5^

Recent studies have shown that mitochondrial dynamics and metabolism contribute broadly to the pathophysiology of human diseases. Alterations in mitochondrial dynamics disrupt different pathways, including energy metabolism and redox balance, leading to bioenergetic dysfunction. Such alterations are implicated not only in cancer but also in other diseases like cardiovascular disorders, highlighting mitochondrial dynamics as a shared pathogenic mechanism. Mitochondrial dynamics have been shown to influence the initiation, metastasis, survival, and stem-like properties of cancer cells, indicating that targeting mitochondrial homeostasis could serve as a potential therapeutic approach in cancer treatment.^24^

This review attempts to offer a detailed framework of the role of mitochondrial energetics and dynamics in TNBC progression, emphasizing the molecular mechanisms underlying the dysregulation of mitochondrial function.

## Mitochondrial dynamics in TNBC

### Mitochondrial fission and fusion machinery

**Figure 1:**
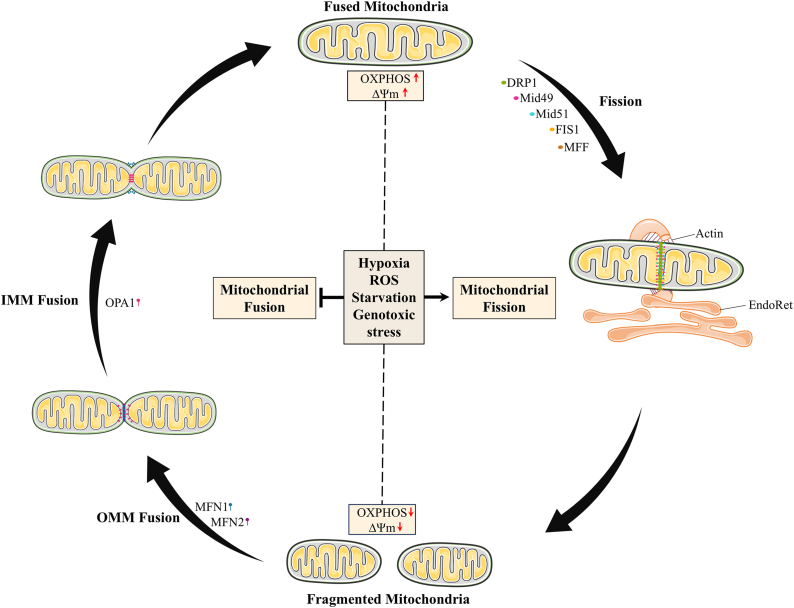
Diagram illustrating the mechanism of mitochondrial dynamics (fission and fusion), along with their pivotal regulators. GTPases MFN1, MFN2, and OPA1 regulate fusion of mitochondria. Mitofusins (MFNs) facilitate outer mitochondrial membrane (OMM) fusion, and OPA1 facilitates inner mitochondrial membrane (IMM) fusion. DRP1 regulates mitochondrial fission.

Mitochondrial dynamics are orchestrated by a group of dynamin-related GTPases that regulate events of fusion and fission (Table 1). DRP1 is the key mediator of mitochondrial fission that is recruited from the cytosol to the outer mitochondrial membrane at the contact site of EndoRet (Endoplasmic Reticulum) and mitochondria by adaptor proteins like mitochondrial dynamics proteins of 49 and 51 kDa (MiD49 and MiD51), mitochondrial fission factor (MFF), and fission 1 (FIS1).^25^ These proteins activate and recruit the DRP1 at the site of action. DRP1 oligomerizes to form a contractile ring around the mitochondria, leading to membrane constriction and division.^25^ Mitofusins (MFN1 and MFN2) drive the fusion of the outer mitochondrial membrane, and OPA1 facilitates the fusion of the inner mitochondrial membrane.^16^ The tightly regulated balance between these two dynamic processes maintains the homeostasis of mitochondria and facilitates responses to cellular stress (Fig. 1).

**Table 1:** Key proteins involved in mitochondrial dynamics.

Protein	Function	Location
DRP1	Fission	Cytosol/outer mitochondrial membrane
MFF	Fission adaptor	Outer mitochondrial membrane
MiD49/51	Fission adaptor	Outer mitochondrial membrane
FIS1	Fission adaptor	Outer mitochondrial membrane
MFN1/2	Fusion	Outer mitochondrial membrane
OPA1	Fusion	Inner mitochondrial membrane

### Dysregulation of mitochondrial dynamics in TNBC

In TNBC, dysregulated mitochondrial dynamics lead to mitochondrial dysfunction and tumor progression. Bhadane et al have reported that in TNBC, the expression of DRP1 is considerably elevated in comparison to other BC subtypes and normal breast tissues.^26^ Knockdown of DRP1 in TNBC suppresses cell proliferation, migration, and invasion, and stimulates apoptosis. Mechanistically, DRP1-mediated mitochondrial fission promotes TNBC growth and metastasis by Ras/rapidly accelerated fibrosarcoma (Raf)/mitogen-activated protein kinase kinase (MEK)/extracellular signal-regulated kinase (ERK) signaling pathway activation.^27^ Seo et al showed that in TNBC, MFF, a DRP1 adaptor protein, was up-regulated and correlated with lower survival rates of patients. Depletion of MFF in TNBC cells suppressed mitochondrial fission, increased OXPHOS, and sensitized cells to chemotherapy. The authors further demonstrated that MFF-induced mitochondrial fission promoted TNBC stemness and chemoresistance by activating the Notch signaling pathway.^28^ Dysregulation of mitochondrial adaptor proteins, including MFF, has been reported across various cancers, where it drives metabolic plasticity and enables tumor cells to adapt to altered energetic demands. For instance, in multiple myeloma, increased expression of MFF induces mitochondrial fission and supports rapid proliferation by enhanced OXPHOS and ATP production.^29^ In BC, aberrant MFF overexpression promotes excessive mitochondrial fission and stimulates a metabolically quiescent phenotype characterized by suppression of mitochondrial biogenesis, OXPHOS, and TCA cycle activity, resulting in reduced ATP levels and impaired energetic efficiency. Additionally, dysregulation of MFF not only facilitates tumor progression and metastasis but also contributes to reduced cancer stem cell populations, which are marked by suppressed mammosphere formation capacity and lower expression of stemness markers, such as aldehyde dehydrogenase (ALDH), leading to increased adaptability and chemoresistance. Similarly, other adaptor proteins, such as FIS1 and Mid49/51, influence mitochondrial architecture and bioenergetics. Together, these studies highlighted the role of adaptor proteins like MFF as crucial mediators of mitochondrial dynamics and metabolic reprogramming, including cancer stem cell regulation.^28^, ^29^, ^30^

The fission of mitochondria links with a decline in mitochondrial membrane potential (ΔΨm), reduced OXPHOS and respiration, a metabolic reprogramming favoring glycolysis, and elevated production of mitochondrial ROS. The degree of oxidative damage, as well as the mitochondrial dynamics, determines the cell’s fate. Mitochondrial ROS contribute to redox signaling, while excessive production of ROS damages cells and may cause cell death.^31^

In contrast to the higher expression of fission proteins, fusion proteins are often down-regulated in TNBC. It has been reported that MFN1 expression is substantially reduced in TNBC in comparison to normal breast tissues. Overexpression of MFN1 in TNBC cell lines suppresses cell proliferation and stimulates apoptosis, while silencing MFN1 produces the opposite effects. The tumor-suppressive function of MFN1 is mediated by its ability to promote OXPHOS and suppress glycolysis.^32^ Similarly, in TNBC, the expression of OPA1 also decreases, and its down-regulation is linked with the increase in metastatic potential and unfavorable clinical results.^33^ Overexpression of OPA1 in TNBC cells inhibits migration and invasion and sensitizes cells to chemotherapy. Mechanistically, OPA1-mediated mitochondrial fusion suppresses TNBC metastasis by inhibiting the epithelial–mesenchymal transition and reducing matrix metalloproteinase (MMP) activity.^33^

Recent studies have highlighted that epigenetic mechanisms, particularly those mediated by non-coding RNAs (ncRNAs), play a major role in the dysregulation of mitochondrial dynamics regulators in various cancers, including TNBC. Different classes of ncRNAs can regulate mitochondrial dynamics at different levels, influencing cellular metabolism, energy production, and mitochondrial homeostasis, which ultimately affects tumor cell survival and chemoresistance. For instance, microRNA-195 (miR-195) acts as an important modulator of mitochondrial dynamics by directly targeting the 3′ UTR of MFN2, which leads to decreased MFN2 expression and increased DRP1 expression, ultimately promoting mitochondrial fission in BC cells. These alterations result in depolarization of mitochondrial membrane potential, reduced oxygen consumption, ATP production, and increased oxidative stress, leading to impaired mitochondrial function and tumor cell growth. Similarly, the long non-coding RNA RACGAP1P, found to be up-regulated in hepatocellular carcinoma and BC, stimulates mitochondrial fission through increased phosphorylation of DRP1 at Ser616 and correlates with increased invasiveness of BC cells.^34^

Furthermore, the interplay between oncogenes, tumor suppressors, and mitochondrial dynamics represents a crucial determinant of metabolic behavior and adaptive capacity of cancer cells, including TNBC. Activation of oncogenes such as MYC and the phosphoinositide 3-kinase (PI3K)/protein kinase B (AKT)/mammalian target of rapamycin (mTOR) pathway has been found to stimulate mitochondrial biogenesis and enhance expression of MFF and DRP1 phosphorylation, which supports enhanced OXPHOS and ATP production necessary for the rapid proliferation of tumor cells.^35^ Similarly, hyperactivation of signaling pathways, such as Ras/Raf/MEK/ERK, induces phosphorylation of DRP1 at Ser616 that drives mitochondrial fragmentation and metabolic reprogramming, supporting survival of tumor cells under stress conditions like nutrient deprivation and chemotherapy.^36^ On the other hand, tumor suppressors act as metabolic checkpoints by maintaining mitochondrial homeostasis. For instance, TP53 up-regulates fusion proteins, including MFN2 and OPA1, thereby decreasing ROS accumulation and maintaining mitochondrial integrity, while phosphatase and tensin homolog (PTEN) deficiency promotes DRP1-mediated mitochondrial fission, driving a metabolic shift towards glycolysis and contributing to an aggressive tumor phenotype.^37^ Moreover, breast cancer 1 (BRCA1) deficiency has been found to impair mitophagy, increase mitochondrial ROS production, and stimulate NLRP3 inflammasome activation, contributing to tumor growth and metastasis in breast cancer cells.^38^ These insights highlight a complex interwoven network in which oncogenes and tumor suppressors converge on mitochondrial dynamics, facilitating tumor cell survival, metabolic flexibility, stress endurance, and therapeutic resistance.

Beyond these molecular interactions, mitochondrial dynamics are tightly coordinated with mitochondrial regulatory pathways, particularly mitophagy and mitochondrial biogenesis, to maintain metabolic adaptability in cancers, including TNBC. During stress conditions like nutrient deprivation or chemotherapy, excessive fission can stimulate mitophagy. PTEN-induced kinase 1 (PINK1)/parkin-mediated mitophagy, along with alternative ubiquitin-dependent pathways, selectively removes damaged mitochondria, which suppresses ROS accumulation and preserves mitochondrial integrity and homeostasis. Impaired mitophagy results in the accumulation of dysfunctional mitochondria, altered OXPHOS, increased ROS levels, and elevated metabolic stress, ultimately contributing to therapy resistance and tumor progression.^24^^,^^37^ In contrast, mitochondrial biogenesis driven by peroxisome proliferator-activated receptor-γ coactivator-1α (PGC-1α) replenishes the functional mitochondrial pool, thereby restoring respiratory capacity to support ATP demand. Up-regulated PGC-1α promotes metabolic shift from glycolysis to OXPHOS under stress conditions, allowing tumor cells to survive and proliferate.^35^^,^^37^^,^^38^

These studies emphasize the significance of mitochondrial dynamics in the progression and chemoresistance of TNBC. The up-regulation of proteins responsible for mitochondrial fission (DRP1 and MFF) and down-regulation of proteins that facilitate mitochondrial fusion (MFNs and OPA1) lead to mitochondrial fragmentation, impaired OXPHOS, increased ROS production, and apoptosis resistance, all of which contribute to TNBC aggressiveness. These mitochondrial abnormalities promote TNBC growth, metastasis, stemness, and chemoresistance through various signaling pathways like Ras/Raf/MEK/ERK, Notch, and epithelial–mesenchymal transition. Thus, targeting dysregulated mitochondrial dynamics represents a potential therapeutic strategy for TNBC.

## Mitochondrial energetics in TNBC

### The Warburg effect and glycolysis

Aerobic glycolysis, or the Warburg effect, is a hallmark of cancer metabolism, whereby cancer cells favor glycolysis to produce energy even when oxygen is present.^39^ Such metabolic reprogramming provides cancer cells with biosynthetic precursors for rapid proliferation and confers resistance to apoptosis.^23^ TNBC exhibits a distinct metabolic phenotype characterized by elevated glycolysis and lactate production.^40^ Pyruvate kinase M2 (PKM2), phosphofructokinase 1 (PFK1), hexokinase 2 (HK2), and other glycolytic enzymes are overexpressed in TNBC and contribute to its aggressive behavior.^41^

HK2 and PFK1, the rate-limiting enzymes in glycolysis, and PKM2 are markedly elevated in TNBC compared with normal breast tissues and other BC subtypes and correlate with patient survival. HK2-mediated glycolysis promoted growth and metastasis of TNBC through the activation of the nuclear factor-kappa B (NF-κB) pathway, while PFK1-induced glycolysis promoted TNBC stemness and chemoresistance by activating the adenosine monophosphate-activated protein kinase (AMPK)/mTOR signaling pathway. PKM2-mediated glycolysis promoted TNBC progression by activating the epidermal growth factor receptor (EGFR)/mitogen-activated protein kinase (MAPK) signaling pathway. Knockdown of these enzymes in TNBC cells inhibits cell proliferation, migration, and invasion, and stimulates apoptosis.^42^ Moreover, studies have shown that PKM2 migrates to the nucleus and functions as a transcriptional co-activator that controls the expression of glycolytic enzymes and cell cycle-related genes.^43^

In addition to the up-regulation of glycolytic enzymes, TNBC exhibits increased lactate production and export. Lactate dehydrogenase A (LDHA) converts pyruvate into lactate, while monocarboxylate transporter 4 (MCT4) facilitates the export of lactate from the cell. Both LDHA and MCT4 are overexpressed in TNBC and contribute to its aggressive phenotype.^44^ Lactate accumulation in the tumor microenvironment supports metastasis, angiogenesis, and immunosuppression.^45^ Targeting the glycolytic pathway and lactate metabolism has emerged as a potential approach to treat TNBC.^46^

### Glutamine metabolism

Glutamine is a vital source of carbon and nitrogen in cancer cells.^47^ TNBC exhibits a high glutamine dependency for growth and survival, a phenomenon referred to as “glutamine addiction”.^21^ Glutamine is taken up by the cell through solute carrier family 1 member 5 (SLC1A5) transporter, where glutaminase (GLS) converts it to glutamate.^15^ Then, this glutamate can subsequently be used in the Krebs cycle to support the production and biosynthesis of ATP.^48^ In TNBC cell lines, glutamine sensitivity is more frequent than in others.^49^

In TNBC, SLC1A5 and GLS expression are significantly elevated in comparison to normal breast tissues and different BC subtypes and are linked with poor patient outcomes. SLC1A5-mediated glutamine uptake was required for TNBC growth and survival by maintaining TCA cycle anaplerosis and supporting lipid synthesis.^50^ GLS-mediated glutaminolysis promoted TNBC chemoresistance by activating the nuclear factor erythroid 2-related factor 2 (NRF2) antioxidant pathway.^50^

Glutamine metabolism not only supports TNBC bioenergetics but also regulates epigenetic modifications and gene expression. α-ketoglutarate, derived from glutamine, is found to be a co-substrate for enzymes like ten-eleven translocation (TET) enzymes as well as Jumonji C (JmjC) domain-containing histone demethylases (JHDMs), both of which are α-ketoglutarate-dependent dioxygenases.^51^ These enzymes catalyze DNA and histone demethylation, respectively, and are crucial for modulating gene expression and cell identity.^52^

These studies underscore the crucial role of glutamine metabolism in TNBC growth, survival, and chemoresistance. The up-regulation of SLC1A5 and GLS leads to increased glutamine uptake and catabolism that supports TNBC bioenergetics, biosynthesis, and epigenetic regulation. Targeting the glutamine metabolic pathway can be a promising therapeutic strategy for TNBC.

### Mitochondrial fatty acid oxidation

In addition to OXPHOS, TNBC cells rely on mitochondrial fatty acid oxidation (FAO) for energy production and biosynthesis. Park et al demonstrated that in TNBC, high ATP levels were maintained by FAO, and TNBC cells exhibited up-regulated carnitine palmitoyl transferase 1A (CPT1A) in FAO, compared with ER ^+^ BC cells.^53^ Many reports showed that CPT1 overexpression was closely associated with cancer progression or chemoresistance in different cancers, including TNBC.^54^^,^^55^ Terunuma et al reported that in ER-negative human tumors, acyl-carnitine levels, a critical intermediate in FAO, were comparatively higher than in ER-positive tumors or non-cancerous tissue.^56^

Studies have shown that chemoresistant TNBC cells, such as MDA-MB-231-R, have enhanced FAO, which contributes to chemoresistance in TNBC. Both cell lines showed higher acyl-CoA synthetase 4 (ACSL4) and carnitine palmitoyltransferase 1B (CPT1B) gene expression.^57^^,^^58^ In TNBC cells, elevated FAO stimulates Src autophosphorylation and, in turn, activated Src phosphorylates ETC (electron transport chain) proteins.^53^ Studies further elucidated the mechanisms responsible for increased FAO in TNBC. The authors found that in TNBC, the expression of the transcription factor, MYC, was significantly elevated, and it directly activated the expression of FAO-related genes.^56^^,^^59^ FAO rewires phospholipid biosynthesis by up-regulating long-chain ACSL4 through acetylated signal transducer and activator of transcription 3 (STAT3). It elevates phospholipid synthesis as well as the levels of mitochondrial membrane phospholipid, resulting in increased mitochondrial membrane potential that acts against the mitochondrial apoptotic pathway.^57^

## Oxidative stress and apoptosis resistance in TNBC

### ROS production and antioxidant

Defensive oxidative stress, resulting from the disrupted balance of ROS generation and antioxidant defense, is a common feature of cancer cells.^60^ Moderate ROS levels promote cancer cell survival by up-regulating MMPs, NF-κB, and vascular endothelial growth factor (VEGF)-dependent angiogenesis and metastasis through MAPK/ERK1/2, p38, c-Jun N-terminal kinase (JNK), and PI3K/Akt pathways. Higher ROS concentrations rupture the mitochondrial membrane, cause DNA damage, and activate the caspase cascade within the tumor microenvironment, leading to apoptosis.^61^

TNBC exhibits increased ROS production compared with other BC subtypes, which contributes to its aggressive phenotype and therapeutic resistance.^62^ The elevated ROS levels in TNBC are attributed to several factors, including mitochondrial dysfunction, increased metabolic activity, impaired OXPHOS, and high glycolytic and glutaminolytic activity, along with the stimulation of MAPK, PI3K/Akt, and other oncogenic signaling pathways.^31^^,^^40^^,^^63^

In the BC xenograft model, the immunoregulatory protein B7–H3 suppresses Nrf2 and its target genes, facilitating glucose metabolism reprogramming via ROS-driven stabilization of hypoxia-inducible factor 1-alpha (HIF1α).^64^ In the case of the TNBC subtype, ROS generation in mitochondria and increased expression of oxidative stress-responsive genes support miRNA Let-7 to stimulate the reprogramming of glucose through enhancement of OXPHOS and glycolysis. Redox cycling and ROS generation assist glucose-6-phosphate (G6PD) to induce P-glycoprotein via the Chk2/p53/NF-κB pathway and cause cell cycle arrest in TNBC.^65^ ROS regulates RAS-mediated invasiveness and matrix metalloproteinase-9 (MMP-9) levels in TNBC. ROS-mediated AKT signaling suppresses the p27 activity in MCF-7 cell lines while up-regulating NRF1-regulated cell cycle genes.^66^

TNBC cells up-regulate antioxidant defense mechanisms, including the NRF2 pathway and the glutathione (GSH) system, to mitigate the increased oxidative stress.^67^ TNBC cells exhibit constitutively activated NRF2, elevated GSH levels, and GSH/oxidized glutathione (GSSG) ratios in comparison to other BC subtypes that show resistance to ROS-induced apoptosis, chemotherapy, and radiotherapy.^68^, ^69^, ^70^ The up-regulation of GSH synthesis and regeneration enzymes in different cancer subtypes, including TNBC, is mediated by NRF2, activator protein-1 (AP-1), NF-κB, and other transcription factors.^71^

### Apoptosis resistance mechanisms

Apoptosis is crucial for tissue homeostasis as well as for preventing tumorigenesis. Cancer cells develop mechanisms to evade apoptosis and acquire therapeutic resistance.^72^ In response to apoptotic stimuli, pro-apoptotic B-cell lymphoma 2 (Bcl-2) proteins assemble to create pores in the outer mitochondrial membrane, releasing different apoptogenic factors, such as cytochrome c, into the cytosol, which interacts with apoptotic protease activating factor-1 (Apaf-1), forming an apoptosome that results in caspase-9 stimulation and caspase cascade initiation.^73^

TNBC cells exhibit reduced pro-apoptotic Bcl-2 protein levels and elevated pro-survival Bcl-2 protein levels, which confer resistance to mitochondrial apoptosis.^74^ It has been reported that Bcl-2 is linked to a higher risk of multidrug resistance in TNBC patients, and its inhibition sensitized patients to these chemotherapeutic drugs.^75^ Bcl-2 and Bcl-xL up-regulation in TNBC is driven by various oncogenic signaling pathways like PI3K/Akt, MAPK, and STAT3.^76^ These pathways activate AP-1, NF-κB, and other transcription factors, which interact with the promoter regions of Bcl-2 and Bcl-xL to enhance their expression.^77^^,^^78^ The mTOR gene, a component of PI3K/Akt/mTOR signaling proteins, essential for controlling cell division, cell survival, and drug resistance, is found to be hyperactive in TNBC.^79^^,^^80^ The down-regulation of anti-survival Bcl-2 proteins in TNBC is mediated via epigenetic silencing and post-translational modifications.^81^^,^^82^ For example, the promoter hypermethylation of Bax and Bak genes in TNBC is reported to suppress their expression. The phosphorylation of Bax by pro-survival kinases like PKCζ and Akt also inhibits its pro-apoptotic activity.^83^

According to the studies of copy number alterations, several tumor types exhibit amplification of B-cell lymphoma 2-like 1 (BCL2L1) (3%) and myeloid cell leukemia-1 (MCL1) (10.9%), encoding Bcl-xl and Mcl-1, respectively, while the amplification of genes that encode Bcl-2, Bcl-w, or Bfl-1 is not detected.^84^ The Cancer Genome Atlas (TCGA) data further support the frequent presence of MCL1 and BCL2L1 amplification.^85^^,^^86^ However, the amplification of distinct anti-apoptotic members of the Bcl-2 protein family shows variation across various cancer types.^86^ Li et al found comparatively high levels of MCL1, Bcl-2, and Bcl-xL expression in TNBC. In all the examined TNBC cell lines, most show relatively high expression levels of MCL1, indicating that it may be a crucial pro-survival factor in TNBC.^80^^,^^87^ Reports have shown that Bcl-xl serves as a more reliable marker for predicting therapeutic responses, and it protects cells from genotoxic stress more effectively than Bcl-2 or MCL1.^88^ High amplification of BCL2L1 with 14% and 15% gain was detected in cervical and colorectal cancer, respectively, while amplification of Mcl-1, a family member of Bcl-2, was detected high in different cancers, such as uterine (9%), ovarian (13%), breast (15%), bladder (16%), liver (17%), and lung (20%) cancer. In diffuse large B-cell lymphoma, significant Bcl-2 amplification, with 11% gain, is observed.^86^

Along with the dysregulation of Bcl-2 family proteins, several researchers have reported elevated expression of IAPs (inhibitor of apoptosis proteins) that inhibit caspases as well as disrupt apoptosis in TNBC cells.^89^^,^^90^ The most well-characterized IAPs are X-linked inhibitor of apoptosis protein (XIAP), cellular inhibitor of apoptosis protein 1 (cIAP1), and cIAP2.^91^^,^^92^ IAPs contain baculovirus IAP repeat (BIR) domains, which interact with and inhibit caspases, as well as really interesting new gene (RING) domains, serving as E3 ubiquitin ligases and facilitating the proteasomal breakdown of caspases.^93^^,^^94^ The transcriptional activation of IAPs in TNBC is mediated by NF-κB, which is constitutively active in this BC subtype.^90^^,^^95^ Studies have correlated poor patient outcomes with the up-regulated expression of IAPs in TNBC.^89^^,^^96^

The aforementioned studies highlight the complex interaction of oxidative stress and apoptosis resistance in TNBC. The increased ROS production, coupled with the up-regulation of antioxidant defense mechanisms and anti-apoptotic proteins, allows TNBC cells to endure oxidative stress and evade apoptosis. The elevated ROS levels activate MAPK, PI3K/Akt, and other oncogenic signaling pathways, which in turn, up-regulate different IAPs (XIAP, cIAP1/2) and pro-survival Bcl-2 proteins like Bcl-xL. Targeting the redox balance and apoptotic machinery offers a promising treatment strategy to overcome TNBC chemoresistance and radioresistance.

## Therapeutic targeting of mitochondrial function in TNBC

### Mitochondrial dynamics modulators

Given the peculiar role of mitochondrial dynamics in TNBC progression and chemoresistance, targeting the fission and fusion machinery has emerged as a promising therapeutic approach.^97^ Several small-molecule inhibitors of DRP1, such as mitochondrial division inhibitor 1 (Mdivi-1), dynasore, and P110, have been demonstrated to suppress fission of mitochondria and trigger apoptosis in various cancer cell lines.^98^ The small molecule Mdivi-1 has been identified as a specific activator of MFN2, which promotes mitochondrial fusion and suppresses tumor growth. Mdivi-1 induces elongation of mitochondria, increases OXPHOS, suppresses the proliferation, migration, and invasion of TNBC cells, and sensitizes cells to chemotherapy.^99^ In a TNBC mouse xenograft model, combining Mdivi-1 either with the chemotherapeutic agent paclitaxel^27^^,^^100^ or doxorubicin^99^^,^^101^ decreases the progression of tumor and metastatic potential of cancer cells. Mdivi-1 can also modulate mitochondrial function and oxidative stress independently of DRP1. While it has been extensively used as a DRP1 inhibitor, its specificity remains limited, as it poorly suppresses DRP1 GTPase activity. To address these limitations, new synthetic inhibitors such as Drpitor 1 and Drpitor1a have been developed, exhibiting improved selectivity and potency towards DRP1. Both compounds demonstrate greater efficacy than Mdivi-1, resulting in reduced proliferation, increased apoptosis, and suppressed tumor growth in cancer cells through the direct inhibition of DRP1-mediated mitochondrial fission. Drpitor1a has also been shown to suppress mitochondrial ROS production and prevent mitochondrial fission.^102^^,^^103^ Another DRP1 inhibitor, dynasore, enhances the cytotoxic effects of cisplatin in TNBC cells (Table 2). The authors further demonstrated that the combination of dynasore and cisplatin induces mitochondrial fusion, increases ROS production, and triggers apoptosis in TNBC cells.^104^ Recent studies have revealed that the bromodomain and extra-terminal domain (BET) inhibitors exert potent anti-tumor effects in TNBC by inducing cell death through modulation of mitochondrial dynamics. It down-regulates the anti-apoptotic protein Bcl-2 and key fission proteins, including DRP1 and MFF, while causing minimal changes in fusion proteins such as OPA1 and MFN2. This results in elongated mitochondria, reduced fragmentation, and improved mitochondrial function, as indicated by increased mitochondrial DNA content and membrane potential. Additionally, combining BET inhibitors with metformin further suppresses TNBC cell growth by targeting the epigenetic–mitochondrial axis.^105^

**Table 2:** Mitochondrial dynamics modulators in preclinical/clinical models of triple-negative breast cancer.

Modulator	Target	Status	Effect	Reference
Mitochondrial division inhibitor (Mdivi-1)	DRP1	Preclinical	Suppresses mitochondrial fission and oxidative phosphorylation; reduces metastasis and induces mitochondrial elongation, and sensitizes cells to chemotherapy	99,107
5-(4-hydroxyphenyl)-3H-1,2-dithiole-3-thione (ADT-OH)	DRP1	Preclinical	Inhibits mitochondrial fission and suppresses migration and invasion; anti-metastatic; triggers the elongation of mitochondria	108
IR-783	DRP1	Preclinical	Promotes DRP1-mediated mitochondrial fission and results in loss of mitochondrial membrane potential and ATP depletion; suppresses tumor growth and induces apoptosis	109
Cepharanthine	DRP1	Preclinical	Cepharanthine/epirubicin combination stimulates the fission of mitochondria and leads to apoptosis by dephosphorylation and mitochondrial translocation of DRP1	110
Dynasore	DRP1	Preclinical	Increases ROS production and triggers apoptosis	111
Silibinin	DRP1	Preclinical	Stimulates mitochondrial fission by increasing DRP1 expression at high concentrations (150–250 μM), resulting in the apoptosis of different cancer cells, including MDA-MB-231; low silibinin concentrations (30–90 μM) promote mitochondrial fusion, leading to the decreased migratory and invasive potential of triple-negative breast cancer cells	112, 113, 114
MYLS22	OPA1	Preclinical	Inhibits mitochondrial fusion and oxidative phosphorylation and suppresses tumor regrowth after chemotherapy	115
Leflunomide	MFN	Clinical	Promotes mitochondrial fusion and sensitizes chemoresistant cells	116,117
Cisplatin	MFN	Clinical	Increases ROS production and triggers apoptosis	118

Beyond their anti-cancer potential, mitochondrial fission inhibitors have also shown promise in mitigating chemotherapy-induced side effects, such as cardiotoxicity. Excessive mitochondrial fission, mediated by DRP1 overactivation, contributes to mitochondrial dysfunction, oxidative stress, and apoptosis in cardiomyocytes exposed to doxorubicin. Studies have revealed that the pharmacological inhibition of this process by agents like Mdivi-1 helps preserve mitochondrial integrity, maintain ATP production, and reduce ROS accumulation. Similarly, newer selective DRP1 inhibitors that prevent its activation or translocation to mitochondria further protect cardiac cells from mitochondrial damage and apoptosis. Thus, targeting mitochondrial dynamics not only improves therapeutic outcomes but also protects against the adverse effects of chemotherapy, such as cardiotoxicity.^106^

### Glycolysis inhibitors

**Figure 2:**
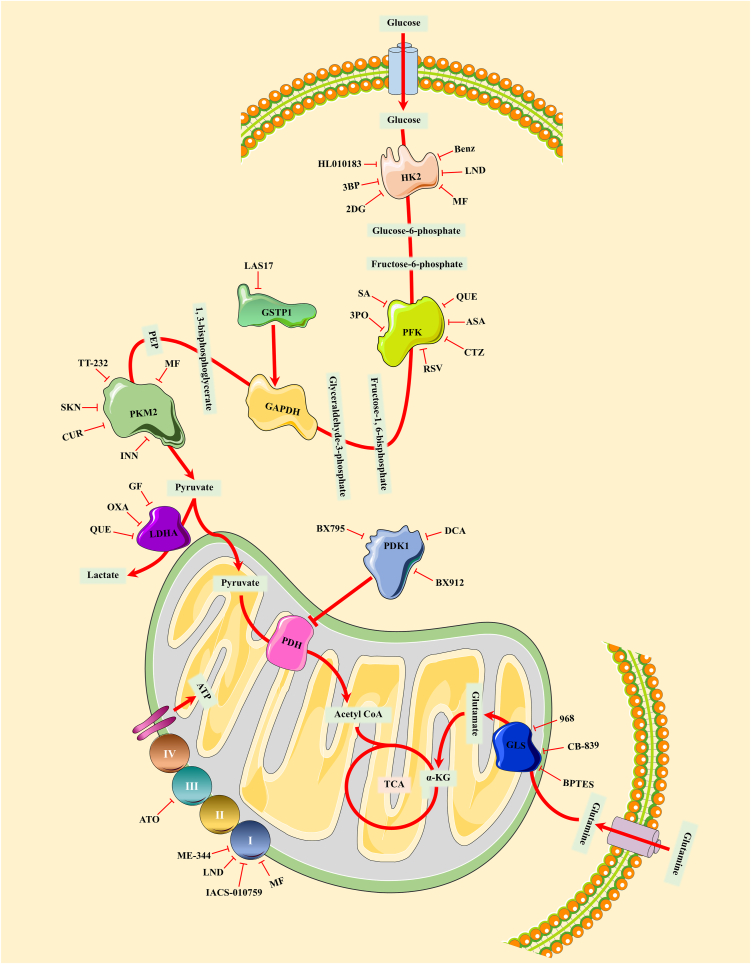
Comprehensive overview of triple-negative breast cancer (TNBC) therapeutics, focusing on the key molecular targets linked with TNBC and the inhibitors being explored in clinical/preclinical trials. The figure represents the key metabolic pathways in TNBC and highlights various points of targeted therapeutic intervention. The metabolic reprogramming in TNBC, characterized by enhanced glycolysis and glutamine metabolism, is depicted with emphasis on the enzymes involved, along with corresponding inhibitors. Glucose is taken up by the cell, and then, HK2 phosphorylates it to produce glucose-6-phosphate in the glycolytic pathway. Inhibitors, such as 2-deoxyglucose (2DG), 3-bromopyruvate (3BP), and lonidamine (LND), specifically target HK2 to suppress glycolytic flux. The formation of fructose-1,6-bisphosphate from fructose-6-phosphate is catalyzed by the enzyme PFK. Inhibitors like salicylic acid (SA), quercetin (QUE), acetylsalicylic acid (ASA), clotrimazole (CTZ), and resveratrol (RSV) target this enzyme to restrict glycolysis further. Downstream, PKM2 mediates the last glycolytic steps, generating pyruvate that is either converted to lactate by LDHA or shunted into mitochondrial metabolism. The figure also highlights the role of mitochondrial metabolism in TNBC. PDH facilitates pyruvate to enter the TCA cycle, and the process is tightly regulated by PDK1. Additionally, the TCA cycle fuels oxidative phosphorylation, a critical energy source for TNBC cells. Inhibitors like ME-344, LND, metformin (MF), and atovaquone (ATO) target different mitochondrial complexes of ETC, disrupting mitochondrial function and energy production. The importance of glutamine metabolism is also emphasized. Glutaminase (GLS) converts glutamine to glutamate, which provides energy for the Krebs cycle and supports biosynthetic processes in TNBC. These strategies highlight the metabolic vulnerabilities of TNBC cells and offer insights into potential therapeutic approaches aimed at disrupting tumor-specific metabolic dependencies.

The elevated glycolytic activity in TNBC provides an attractive opportunity for therapeutic targeting.^119^^,^^120^ In various cancer models, several glycolytic inhibitors like lonidamine (LND), 3-bromopyruvate (3BP), and 2-deoxyglucose (2DG) have been developed and have demonstrated the potential to suppress the growth of tumors and metastasis.^121^^,^^122^ Treatment with 2DG inhibits cell proliferation, triggers apoptosis, and makes TNBC cells more sensitive to chemotherapy.^123^^,^^124^ Combining 2DG and the chemotherapeutic agent, docetaxel, significantly decreases tumor progression and metastatic potential in a TNBC mouse xenograft model.^125^ Similarly, 3BP treatment inhibits glycolysis, increases ROS production, and triggers apoptosis in TNBC cells.^126^ The authors further demonstrate that the combination of 3BP with the chemotherapeutic agent doxorubicin significantly reduces the progression of tumor and improves the survival rate in mouse models (Fig. 2). The combination of 2DG and 3BP is reported to enhance the efficiency of photodynamic therapy in BC cells.^121^^,^^127^

In addition to the direct inhibition of glycolytic enzymes, targeting the upstream regulators of glycolysis has also shown promise in TNBC treatment. The transcription factor HIF1α serves as a critical regulator of glycolysis and is often up-regulated in TNBC.^128^ HIF1α inhibition by small molecule inhibitors, such as PX-478 and acriflavine, suppresses glycolysis, induces apoptosis, and sensitizes TNBC cells to chemotherapy.^129^ The combination of HIF1α inhibitors with chemotherapeutic agents markedly decreases metastasis and the growth of tumors.^130^^,^^131^ Another upstream regulator of glycolysis that is constitutively active in TNBC is the PI3K/Akt/mTOR pathway.^130^^,^^132^ The inhibition of this pathway by small molecule inhibitors, such as BKM120 and everolimus, suppresses glycolysis, induces apoptosis, and sensitizes TNBC cells to chemotherapy.^79^^,^^133^ The combination of PI3K/Akt/mTOR inhibitors with chemotherapeutic molecules also synergistically reduces the progression of tumors and metastatic potential in TNBC mouse models.^79^^,^^133^

### GLS inhibitors

Glutamine, a crucial anaplerotic substrate for the citric acid cycle, is important especially for rapidly dividing cells, including neoplastic cells. It acts as a precursor for GSH, an antioxidant that is essential to regulate cellular redox balance homeostasis and protect cells from oxidative damage. Disturbance in glucose metabolism leads to stimulation of oxidative metabolism by glutamine.^134^^,^^135^ The dependence of TNBC on glutamine metabolism has led to the exploration of GLS inhibitors as a potential treatment approach.^136^ A highly selective and potent GLS inhibitor, CB-839, has demonstrated anti-cancer activity in various cancer models.^137^^,^^138^ In TNBC, CB-839 treatment inhibits cell proliferation, induces apoptosis, and sensitizes cells to chemotherapy. The combination of CB-839 with the chemotherapeutic agent paclitaxel significantly inhibits metastatic potential and the growth of tumors in a TNBC mouse xenograft model.^139^^,^^140^ Lampa et al have shown that combining CB-839 and the mTOR inhibitor everolimus induces synthetic lethality in TNBC cells.^141^ Hong et al have demonstrated that the combination of CB-839 with carboplatin substantially delays tumor progression and increases survival in TNBC.^142^ Inhibition of glutamine metabolism and mTOR signaling leads to a severe depletion of TCA cycle intermediates, increased ROS production, and apoptosis induction in TNBC cells.^143^

Targeting the glutamine transporter SLC1A5 in addition to GLS inhibitors has also demonstrated potential in TNBC treatment. The compound V-9302 is a specific inhibitor of SLC1A5 that suppresses glutamine uptake and inhibits tumor growth in several cancer models.^144^^,^^145^ In TNBC, V-9302 treatment inhibits cell proliferation, induces apoptosis, and sensitizes cells to chemotherapy.^146^ The combination of V-9302 with the anti-PDL1 antibody strongly inhibits tumor growth in TNBC cells.^147^ Compound 968 is another inhibitor that can limit the growth of tumors and cancer cell proliferation in TNBC and other cancers (Fig. 2). The combination of 968 with the small molecule inhibitor of the enzyme GLS, bis-2-(5-phenylacetamido-1,3,4-thiadiazol-2-yl) ethyl sulphide (BPTES), synergistically reduces the growth of tumors in TNBC cells.^148^^,^^149^

### Mitochondrial fatty acid oxidation inhibitors

The enhanced FAO in TNBC presents a promising opportunity for therapeutic strategies.^57^ In different cancer models, including TNBC, many FAO inhibitors (etomoxir, perhexiline, oxfenicine, trimetazidine, etc.) have been developed that can reduce tumor growth and metastasis (Fig. 2). Inhibition of CPT1A, a rate-limiting enzyme in FAO, with the small molecule inhibitor, etomoxir, has been shown to suppress FAO and selectively reduce TNBC cell growth and proliferation and induce apoptosis (Table 3). A significantly elevated phosphorylated AMPK level, a key bioenergetic stress marker, has also been shown in tumors treated with etomoxir compared with the control.^54^^,^^150^ Sirois et al found that chemoresistant TNBC cells were more susceptible to etomoxir treatment.^151^

**Table 3:** List of therapeutic molecules targeting mitochondrial function in clinical/preclinical triple-negative breast cancer (TNBC) models.

Drugs	Target	Status	Effect	Reference
TT-232	PKM2	Clinical	It inhibits the proliferation of different cancer cells and stimulates apoptosis.	155,156
Shikonin	PKM2	Preclinical	Shikonin and its analogs, such as alkannin, diminish glycolysis and lactate production in drug-sensitive and drug-resistant cell lines.	157
Lapatinib	PKM2	Preclinical	It inhibits cell proliferation by reducing PKM2 expression.	158
Curcumin	PKM2	Clinical	It suppresses PKM2 by mTOR–HIF1α axis inhibition.	159
			It reduces cell viability, glucose uptake, and lactate release.	
BX795 and BX912	PDK1	Preclinical	It lowers the viability of different TNBC cell lines that express MYC, including Hs578T and MDA-MB-231; in the MDA-MB-231 cell line, it reduces the CD44^+^/CD24-population.	160
Dichloroacetate	PDK1	Clinical	It inhibits the metastatic breast cancer cell growth in both *in vitro* and *in vivo* models.	161
Quercetin	PFK	Preclinical	It affects the expression of PFKP (phosphofructokinase platelet-type) and lactate production in MDA-MB-231 cells; it suppresses aerobic glycolysis, migration, and metastasis.	162
	LDHA		It affects the PFKP-LDHA pathway and hinders the increased migration of breast cancer cells mediated through aerobic glycolysis.	162
Clotrimazole	PFK	Preclinical	It suppresses PFK activity by dissociating it from F-actin, leading to impaired glycolysis in human breast cancer tissues.	163, 164, 165
			It reduces cell proliferation, rates of glucose consumption, mitochondrial activity, and ATP production in different cancer cell lines, including MCF-7 and MDA-MB-231.	
			It induces a phenotype-dependent suppression of HK, PK, and PFK-1.	
Acetylsalicylic acid and salicylic acid	PFK	–	Both suppress PFK activity, decrease glucose utilization, and diminish cell viability in MCF-7 cell lines.	166
PFKFB3 inhibitors	PFK	Preclinical	3PO reduces the PFK-2 activity of the isozyme PFKFB3 at low fructose-6-phosphate concentrations; it reduces tumor growth in the MDA-MB-231 xenograft model and diminishes cell viability and glycolysis in breast cancer cell lines. It also aids in the enhanced production of lactate due to the increased glutamine oxidation.	167
Resveratrol	PFK	Preclinical	It directly suppresses PFK activity, reducing cell viability, cellular ATP content, and glucose consumption in MCF-7 cell lines.	168
2-deoxyglucose	HK2	Clinical	It suppresses the proliferation of cells, triggers apoptosis, and sensitizes cells to chemotherapy.	123, 124, 125
			Combination therapy of 2-deoxyglucose and docetaxel significantly decreases the growth of the tumor and metastasis in a TNBC mouse xenograft model.	
3-bromopyruvate	HK2	Preclinical	It induces oxidative stress in MDA-MB-231 cell lines by decreasing glutathione levels and the activities of antioxidant enzymes, which leads to apoptosis.	121,127,169,170
			Combination treatment of 3-bromopyruvate and methyl jasmonate markedly reduces tumors in the TNBC xenograft model.	
			A combination of 2-deoxyglucose and 3-bromopyruvate suppresses cell adhesion and migration and enhances the efficiency of photodynamic therapy in breast cancer cells.	
Benserazide	HK2	Preclinical	It decreases anaerobic glycolysis and suppresses tumor growth in breast cancer cells.	171
HL010183	HK2	Preclinical	Derivative of metformin. It suppresses the growth of tumor, proliferative, and invasive properties in MDA-MB-231 xenograft models.	172
Metformin	HK2	Clinical trials to repurpose	It inhibits TNBC stem cells and lowers the capacity to induce tumors in different TNBC models, including xenograft models and the MDA-MB-231 cell line.	173,174
	PKM2		It decreases the expression of PKM2, suppresses the proliferation of cells, and stimulates apoptosis only in nutrient-poor conditions. Apoptosis induction is mediated by Stat3 inactivation.	174, 175, 176
	Mitochondrial complex I		It suppresses TNBC cell proliferation via AMPK induction by blocking mitochondrial complex 1, inhibiting mTOR.	177
Lonidamine	HK2	Preclinical	It stimulates acidification of the intracellular tumor, causes ATP depletion, and sensitizes breast cancer cells to doxorubicin.	178
	Mitochondrial complex I		It promotes apoptosis of breast cancer cells through ROS overproduction and depolarization of the mitochondrial membrane.	179
ME-344	Mitochondrial complex I	Clinical	In the MMTV-PyMT (mouse mammary tumor virus-polyoma middle tumor-antigen) model, it makes breast cancer cells more susceptible to inhibitors of tyrosine kinases.	180
IACS-010759	Mitochondrial complex I	Clinical	It suppresses the cell viability and growth of TNBC cell lines and other breast cancer cell lines, while leaving normal cells unaffected.	181
Atovaquone/AINRs	Mitochondrial complex III	Clinical trials to repurpose	It inhibits oxidative phosphorylation by suppressing mitochondrial complex III, leading to a reduced consumption of endogenous oxygen and intracellular ATP in 4T1 and MDA-MB-231 cell lines.	182
LAS17	Glutathione S-transferase Pi 1 (GSTP1)	Preclinical	It inhibits tumor growth and the survival potential of cells in the TNBC xenograft model.	183
CB-839	Glutaminase	Clinical	It suppresses the proliferation of breast cancer cells, triggers apoptosis, and sensitizes cells to chemotherapy.	137, 138, 139, 140,142
			Combination treatment of CB-839 and paclitaxel significantly decreases the progression of tumor and metastatic potential in the TNBC mouse xenograft model.	
			Combining CB-839 with carboplatin delays the progression of the tumor and increases the survival rate in the mouse xenograft model of TNBC.	
968 and BPTES	Glutaminase	–	968 suppresses tumor progression and proliferation of different cancers.	148,149
			Combination treatment of compound 968 and BPTES synergistically decreases tumor growth in TNBC cells.	
V-9302	SLC1A5	Preclinical	It reduces glutamine uptake and tumor growth in different cancer models; inhibits cell proliferation, stimulates apoptosis, and sensitizes cells to chemotherapy.	144, 145, 146, 147
			A combination of V-9302 and anti-PDL1 antibody significantly suppresses tumor growth in TNBC cells.	
Oxamate	LDHA	Preclinical	It decreases cellular lactate production, inhibits cell proliferation, and reduces tumor growth in TNBC.	184,185
Galloflavin	LDH	Preclinical	It inhibits the expression of LDH, stimulates oxidative stress, and decreases cell proliferation in the MDA-MB-231 cell line.	186,187
Etomoxir	CPT1	Preclinical	It suppresses FAO, inhibits the growth and proliferation of cells, and triggers apoptosis in TNBC.	54,152
			Etomoxir and GSK126 combination synergistically reduces the progression of tumors in the TNBC xenograft model.	
Perhexiline	CPT1	Clinical	It inhibits tumor growth and cancer stem cell population in MMTV-PyMT tumors.	55

The combination of etomoxir with GSK126 synergistically suppresses tumor growth in the TNBC xenograft model.^152^ Moreover, etomoxir alone or in combination with bexarotene suppresses the FAO activity completely.^153^ When combined with either mercaptoacetate, which suppresses lipolysis, or orlistat, which suppresses *de novo* fatty acid synthesis, etomoxir produces synergistic effects in different tumors.^154^ In mouse mammary tumor virus-polyoma middle tumor-antigen (MMTV-PyMT) tumors, perhexiline, another CPT1 inhibitor, inhibits tumor growth, expression of Sox2, and cancer stem cell population.^55^ These studies suggest that targeting the FAO may represent a potential approach to treat TNBC.

### Antioxidant inhibitors

The up-regulation of antioxidant defense mechanisms in TNBC represents another potential therapeutic target. The inhibition of NRF2, a key regulator of antioxidant gene expression, makes TNBC cells more sensitive to radiotherapy and chemotherapy.^70^ In various cancer models, inhibition of NRF2 using a specific inhibitor, brusatol, increases the cytotoxic effects of chemotherapeutic agents.^188^^,^^189^ In TNBC, brusatol treatment inhibits NRF2 activity, increases ROS production, and sensitizes cells to chemotherapy.^190^ Chandrasekaran et al have reported that the combined treatment of brusatol with paclitaxel increases ROS production and growth inhibitory activity and decreases the migratory potential of TNBC cells.^191^ In different cancer models, combining brusatol with different chemotherapeutic agents like doxorubicin, etoposide, cisplatin, and 5-fluorouracil synergistically reduces tumor growth and metastasis.^192^^,^^193^ The natural compound pterostilbene significantly suppresses the growth and metastasis of TNBC cells and promotes apoptosis. Pterostilbene also suppresses mTOR and p-AKT and shows a subsequent elevation in Bax protein levels.^194^ It has been reported that in TNBC cells, pterostilbene promotes TNF-related apoptosis-inducing ligand (TRAIL)-induced apoptosis.^195^ While pterostilbene demonstrates chemoreception of TNBC cells, epigallocatechin-3-gallate (EGCG) exhibits chemosensitization of TNBC cells to cisplatin by activating NRF2–ARE signaling.^196^ De Blasio et al have reported that NRF2 promotes proliferation and antioxidant capacity in TNBC cells through decreased miR-29b-1-5p expression. By suppressing N-methyltransferase expression and lowering p-NRF2 and p-AKT levels, miR-29b-1-5p stimulates cytotoxic effects that aid in inhibiting invasion and cell proliferation.^68^

In addition to NRF2 inhibitors, compounds that deplete cellular GSH levels have also shown anti-tumor activity in TNBC. Buthionine sulfoximine selectively inhibits glutamate-cysteine ligase (GCL), resulting in an elevated ROS production, depletion of GSH levels, and sensitization of TNBC cells to chemotherapy and radiotherapy.^197^

### BH3 mimetics

Bcl-2 homology 3 (BH3) mimetics function as inhibitors by binding to pro-survival family members of Bcl-2. This interaction releases BH3-only proteins, disrupting the equilibrium between pro-survival and pro-apoptotic Bcl-2 family proteins, which results in the stimulation of apoptosis.^198^ BH3 mimetics can significantly induce changes in mitochondrial structure, such as discontinuities in the outer mitochondrial membrane and swollen mitochondrial matrix, upstream of activation of caspase.^199^ In cancer, they induce mitochondrial death pathways and are recognized as significant modulators of different cell mechanisms that contribute to poor treatment responses, which include paracrine signaling to the tumor microenvironment and immunological regulation, cancer-specific metabolic pathways, and stemness of cancer cells.^200^

Employing BH3 mimetics alone or combining them with other drugs to target anti-apoptotic proteins of the Bcl-2 family directly shows an effective option to treat different cancers, including colorectal cancer, chronic lymphocytic leukemia, small-cell lung cancer, and multiple myeloma. In TNBC, the overexpression of pro-survival Bcl-2 proteins has led researchers to explore BH3 mimetics as a potential treatment opportunity.^80^ Venetoclax (ABT-199), navitoclax (ABT-263), and ABT-737 are among the most extensively studied BH3 mimetics that inhibit Bcl-xL, Bcl-w, and Bcl-2.^201^ In TNBC, ABT-737 treatment induces apoptosis and sensitizes cells to chemotherapy. ABT-737 and GO-203 (an inhibitor of MUC1-C) together suppress the proliferation of ABT-737 and ABT-263-resistant TNBC cell lines.^202^ Venetoclax (ABT-199) exhibits synergism with cisplatin^88^ and doxorubicin.^203^

Karpel-Massler et al have shown that combining ABT-263 with Gamitrinib G-TPP, a mitochondrial matrix chaperone inhibitor (HSP90 inhibitor), massively activates apoptosis in the preclinical models of different tumors, including TNBC.^204^ The combination of navitoclax with acriflavine, an MCL-1 downregulator, demonstrates enhanced toxic effects in different TNBC cell lines, including MDAMB-231 and HS578T.^205^ The combination of ABT-263 with the ALK/ROS1 inhibitor crizotinib significantly reduces proliferation and promotes a pro-apoptotic stimulus in TNBC cells.^206^ The combination of ABT-263 with PI3K and mTOR inhibitor (NVP-BEZ235) demonstrates a significant synergism against BC cells, including TNBC, even at lower concentrations.^207^

In addition to the Bcl-2 family inhibitors, compounds that target IAPs have also shown promise in TNBC treatment.^208^ It is reported that xevinapant targets XIAP, cIAP1, and cIAP2 and induces apoptosis. Xevinapant has demonstrated potential anti-tumor activities in preclinical studies of different tumors.^89^^,^^209^ The small molecule compound SM-406 selectively inhibits XIAP and cIAP1/2 and triggers apoptosis across different cancers. In TNBC, SM-406 treatment suppresses cell growth, induces apoptosis, and sensitizes cells to chemotherapy.^210^ It has been found that SM-406 has the ability to activate caspases and induce apoptosis in the MDA-MB-231 cells. SM-406 also reduces metastasis and the growth of tumors in TNBC.^211^

## Conclusion and future perspective

TNBC is a highly invasive and metastatic BC subtype, which lacks targeted therapies and relies on chemotherapy as the mainstay of treatment. The development of chemoresistance poses a significant challenge in managing TNBC and contributes to the poor prognosis of the disease. Studies suggest that mitochondrial dysfunction is crucial in TNBC progression and therapeutic resistance. The dysregulation of mitochondrial dynamics, metabolic reprogramming, oxidative stress, and apoptosis resistance are prominent features of TNBC that contribute to its aggressive phenotype and poor clinical outcomes.

This review highlights the molecular mechanisms behind the dysregulation of mitochondrial function in TNBC and emphasizes the therapeutic potential of targeting these pathways. Promoting mitochondrial fusion, suppressing mitochondrial fission, glycolysis, and glutaminolysis, depleting antioxidant defenses, and activating apoptosis are promising strategies for TNBC treatment. The combination of mitochondrial-targeted agents with conventional chemotherapeutic drugs has demonstrated significant anti-tumor activities in different preclinical models of TNBC.

A key challenge in targeting mitochondrial function in TNBC is the high degree of heterogeneity within this subtype. The most aggressive and chemoresistant TNBC subtype is the basal-like subtype. The mesenchymal and LAR subtypes also have distinct metabolic and apoptotic signatures. So, exploring the heterogeneity of TNBC and identifying predictive biomarkers is crucial for personalized treatment strategies. Another challenge in targeting mitochondrial function in TNBC is the potential toxicity to normal tissues that rely on mitochondrial metabolism, especially the heart, brain, and skeletal muscle. The use of nanoparticle-based drug delivery systems and antibody-drug conjugates may help to overcome this challenge by selectively targeting mitochondrial inhibitors to TNBC cells.

Finally, the dynamic interaction between mitochondrial function and the tumor microenvironment in TNBC requires further investigation. The increased glycolytic activity and lactate production in TNBC cells can create an acidic and immunosuppressive microenvironment that promotes tumor growth and metastasis. Targeting mitochondrial metabolism may not only have direct cytotoxic effects on TNBC cells but also regulate the tumor microenvironment and boost anti-tumor immunity. The combination of mitochondrial inhibitors with immunotherapeutic agents, such as immune checkpoint inhibitors, could provide a novel approach to treating TNBC.

Targeting mitochondrial function represents a potential treatment approach for TNBC, showing significant anti-cancer effects in different preclinical studies of TNBC. However, the clinical translation of these findings requires further investigation in well-designed clinical trials. With advancements in mitochondrial biology and novel drug delivery systems, the targeting of mitochondrial function may open new possibilities for personalized TNBC treatment in the future.

## CRediT authorship contribution statement

**Harshit Mishra:** Writing – review & editing, Writing – original draft, Resources, Conceptualization. **Anshu Yadav:** Writing – review & editing, Writing – original draft, Resources. **Veena B. Kushwaha:** Writing – review & editing, Supervision. **Manish Pratap Singh:** Writing – review & editing, Writing – original draft, Supervision, Resources, Conceptualization.

## Conflict of interests

The authors declare that they have no known competing financial interests or personal relationships that could have appeared to influence the work reported in this paper.

## References

[1] Sung H., Ferlay J., Siegel R.L. (2021). Global cancer statistics 2020: GLOBOCAN estimates of incidence and mortality worldwide for 36 cancers in 185 countries. CA Cancer J Clin.

[2] Shaath H., Elango R., Alajez N.M. (2021). Molecular classification of breast cancer utilizing long non-coding RNA (lncRNA) transcriptomes identifies novel diagnostic lncRNA panel for triple-negative breast cancer. Cancers.

[3] Yin L., Duan J.J., Bian X.W., Yu S.C. (2020). Triple-negative breast cancer molecular subtyping and treatment progress. Breast Cancer Res.

[4] Silvestri M., Dugo M., Vismara M. (2022). Copy number alterations analysis of primary tumor tissue and circulating tumor cells from patients with early-stage triple negative breast cancer. Sci Rep.

[5] Gong Y., Ji P., Yang Y.S. (2021). Metabolic-pathway-based subtyping of triple-negative breast cancer reveals potential therapeutic targets. Cell Metab.

[6] Verma A., Singh A., Singh M.P. (2022). EZH2-H3K27me3 mediated KRT14 upregulation promotes TNBC peritoneal metastasis. Nat Commun.

[7] O’Reilly D., Al Sendi M., Kelly C.M. (2021). Overview of recent advances in metastatic triple negative breast cancer. World J Clin Oncol.

[8] Yao Y., Chu Y., Xu B., Hu Q., Song Q. (2019). Risk factors for distant metastasis of patients with primary triple-negative breast cancer. Biosci Rep.

[9] Jin J., Gao Y., Zhang J. (2018). Incidence, pattern and prognosis of brain metastases in patients with metastatic triple negative breast cancer. BMC Cancer.

[10] Lehmann B.D., Pietenpol J.A. (2014). Identification and use of biomarkers in treatment strategies for triple-negative breast cancer subtypes. J Pathol.

[11] Liu Y.R., Jiang Y.Z., Xu X.E. (2016). Comprehensive transcriptome analysis identifies novel molecular subtypes and subtype-specific RNAs of triple-negative breast cancer. Breast Cancer Res.

[12] Li Y., Zhan Z., Yin X., Fu S., Deng X. (2021). Targeted therapeutic strategies for triple-negative breast cancer. Front Oncol.

[13] Zhang B., Pan C., Feng C. (2022). Role of mitochondrial reactive oxygen species in homeostasis regulation. Redox Rep.

[14] Lee Y.G., Park D.H., Chae Y.C. (2022). Role of mitochondrial stress response in cancer progression. Cells.

[15] Sun X., Wang M., Wang M. (2020). Metabolic reprogramming in triple-negative breast cancer. Front Oncol.

[16] Weiner-Gorzel K., Murphy M. (2021). Mitochondrial dynamics, a new therapeutic target for triple negative breast cancer. Biochim Biophys Acta Rev Cancer.

[17] Wang S., Zhao H., Lin S. (2023). New therapeutic directions in type II diabetes and its complications: mitochondrial dynamics. Front Endocrinol.

[18] Adebayo M., Singh S., Singh A.P., Dasgupta S. (2021). Mitochondrial fusion and fission: the fine-tune balance for cellular homeostasis. FASEB J.

[19] Srinivasan S., Guha M., Dong D.W. (2016). Disruption of cytochrome c oxidase function induces the Warburg effect and metabolic reprogramming. Oncogene.

[20] Hanahan D., Weinberg R.A. (2011). Hallmarks of cancer: the next generation. Cell.

[21] Quek L.E., van Geldermalsen M., Guan Y.F. (2022). Glutamine addiction promotes glucose oxidation in triple-negative breast cancer. Oncogene.

[22] Liu C., Jin Y., Fan Z. (2021). The mechanism of Warburg effect-induced chemoresistance in cancer. Front Oncol.

[23] Liberti M.V., Locasale J.W. (2016). The Warburg effect: how does it benefit cancer cells?. Trends Biochem Sci.

[24] Rocca C., Soda T., De Francesco E.M. (2023). Mitochondrial dysfunction at the crossroad of cardiovascular diseases and cancer. J Transl Med.

[25] Kraus F., Roy K., Pucadyil T.J., Ryan M.T. (2021). Function and regulation of the divisome for mitochondrial fission. Nature.

[26] Bhadane D., Kamble D., Deval M., Das S., Sitasawad S. (2024). NOX4 alleviates breast cancer cell aggressiveness by co-ordinating mitochondrial turnover through PGC1α/Drp1 axis. Cell Signal.

[27] Zhao J., Zhang J., Yu M. (2013). Mitochondrial dynamics regulates migration and invasion of breast cancer cells. Oncogene.

[28] Seo J.H., Chae Y.C., Kossenkov A.V. (2019). MFF regulation of mitochondrial cell death is a therapeutic target in cancer. Cancer Res.

[29] Cantafio M.E.G., Valentino I., Torcasio R. (2025). Mitochondrial fission factor drives an actionable metabolic vulnerability in multiple myeloma. Haematologica.

[30] Sánchez-Alvarez R., De Francesco E.M., Fiorillo M., Sotgia F., Lisanti M.P. (2020). Mitochondrial fission factor (MFF) inhibits mitochondrial metabolism and reduces breast cancer stem cell (CSC) activity. Front Oncol.

[31] Punter K.B., Chu C., Chan E.Y.W. (2023). Mitochondrial dynamics and oxidative phosphorylation as critical targets in cancer. Endocr Relat Cancer.

[32] Kannan A., Wells R.B., Sivakumar S. (2016). Mitochondrial reprogramming regulates breast cancer progression. Clin Cancer Res.

[33] Li G., Zhou J., Budhraja A. (2015). Mitochondrial translocation and interaction of cofilin and Drp1 are required for erucin-induced mitochondrial fission and apoptosis. Oncotarget.

[34] Gallo Cantafio M.E., Torcasio R., Viglietto G., Amodio N. (2023). Non-coding RNA-dependent regulation of mitochondrial dynamics in cancer pathophysiology. Noncoding RNA.

[35] Huang A., Xue H., Xie T. (2025). A review of the pathogenesis of mitochondria in breast cancer and progress of targeting mitochondria for breast cancer treatment. J Transl Med.

[36] Singh S.R., Bhaskar R., Ghosh S. (2025). Exploring the genetic orchestra of cancer: the interplay between oncogenes and tumor-suppressor genes. Cancers.

[37] Pei W., Dai L., Li M. (2025). Targeting mitochondrial quality control for the treatment of triple-negative breast cancer: from molecular mechanisms to precision therapy. Biomolecules.

[38] Ruidas B. (2025). Mitochondrial dynamics in breast cancer metastasis: from metabolic drivers to therapeutic targets. Oncol Adv.

[39] Warburg O. (1956). On the origin of cancer cells. Science.

[40] Wang Z., Jiang Q., Dong C. (2020). Metabolic reprogramming in triple-negative breast cancer. Cancer Biol Med.

[41] Wu Z., Wu J., Zhao Q., Fu S., Jin J. (2020). Emerging roles of aerobic glycolysis in breast cancer. Clin Transl Oncol.

[42] Tseng C.W., Kuo W.H., Chan S.H., Chan H.L., Chang K.J., Wang L.H. (2018). Transketolase regulates the metabolic switch to control breast cancer cell metastasis via the α-ketoglutarate signaling pathway. Cancer Res.

[43] Pandkar M.R., Raveendran A., Biswas K. (2023). PKM2 dictates the poised chromatin state of PFKFB3 promoter to enhance breast cancer progression. NAR Cancer.

[44] Dev Arundhathi J.R., Mathur S.R., Gogia A., Deo S.V.S., Mohapatra P., Prasad C.P. (2021). Metabolic changes in triple negative breast cancer-focus on aerobic glycolysis. Mol Biol Rep.

[45] Gao Y., Zhou H., Liu G., Wu J., Yuan Y., Shang A. (2022). Tumor microenvironment: lactic acid promotes tumor development. J Immunol Res.

[46] San-Millán I., Brooks G.A. (2017). Reexamining cancer metabolism: lactate production for carcinogenesis could be the purpose and explanation of the Warburg effect. Carcinogenesis.

[47] Jia D., Lu M., Jung K.H. (2019). Elucidating cancer metabolic plasticity by coupling gene regulation with metabolic pathways. Proc Natl Acad Sci U S A.

[48] Yang L., Venneti S., Nagrath D. (2017). Glutaminolysis: a hallmark of cancer metabolism. Annu Rev Biomed Eng.

[49] Bel’skaya L.V., Dyachenko E.I. (2024). Oxidative stress in breast cancer: a biochemical map of reactive oxygen species production. Curr Issues Mol Biol.

[50] Kim J.H., Lee K.J., Seo Y. (2018). Effects of metformin on colorectal cancer stem cells depend on alterations in glutamine metabolism. Sci Rep.

[51] Wang D., Meng G., Zheng M. (2016). The glutaminase-1 inhibitor 968 enhances dihydroartemisinin-mediated antitumor efficacy in hepatocellular carcinoma cells. PLoS One.

[52] Figueroa M.E., Abdel-Wahab O., Lu C. (2010). Leukemic IDH1 and IDH2 mutations result in a hypermethylation phenotype, disrupt TET2 function, and impair hematopoietic differentiation. Cancer Cell.

[53] Park J.H., Vithayathil S., Kumar S. (2016). Fatty acid oxidation-driven src links mitochondrial energy reprogramming and oncogenic properties in triple-negative breast cancer. Cell Rep.

[54] Camarda R., Zhou A.Y., Kohnz R.A. (2016). Inhibition of fatty acid oxidation as a therapy for MYC-overexpressing triple-negative breast cancer. Nat Med.

[55] Wang T., Fahrmann J.F., Lee H. (2018). JAK/STAT3-regulated fatty acid β-oxidation is critical for breast cancer stem cell self-renewal and chemoresistance. Cell Metab.

[56] Terunuma A., Putluri N., Mishra P. (2014). MYC-driven accumulation of 2-hydroxyglutarate is associated with breast cancer prognosis. J Clin Invest.

[57] Li Y.J., Fahrmann J.F., Aftabizadeh M. (2022). Fatty acid oxidation protects cancer cells from apoptosis by increasing mitochondrial membrane lipids. Cell Rep.

[58] Sinha A., Saini K.K., Chandramouli A. (2024). ACSL4-mediated H3K9 and H3K27 hyperacetylation upregulates SNAIL to drive TNBC metastasis. Proc Natl Acad Sci U S A.

[59] Horiuchi D., Kusdra L., Huskey N.E. (2012). MYC pathway activation in triple-negative breast cancer is synthetic lethal with CDK inhibition. J Exp Med.

[60] Jiang L., Shestov A.A., Swain P. (2016). Reductive carboxylation supports redox homeostasis during anchorage-independent growth. Nature.

[61] Aggarwal V., Tuli H.S., Varol A. (2019). Role of reactive oxygen species in cancer progression: molecular mechanisms and recent advancements. Biomolecules.

[62] Sarmiento-Salinas F.L., Delgado-Magallón A., Montes-Alvarado J.B. (2019). Breast cancer subtypes present a differential production of reactive oxygen species (ROS) and susceptibility to antioxidant treatment. Front Oncol.

[63] Ježek J., Cooper K.F., Strich R. (2018). Reactive oxygen species and mitochondrial dynamics: the Yin and Yang of mitochondrial dysfunction and cancer progression. Antioxidants.

[64] Lim S., Liu H., Madeira da Silva L. (2016). Immunoregulatory protein B7-H3 reprograms glucose metabolism in cancer cells by ROS-mediated stabilization of HIF1α. Cancer Res.

[65] Wang W., Cai Q., Zhou F. (2018). Impaired pentose phosphate pathway in the development of 3D MCF-7 cells mediated intracellular redox disturbance and multi-cellular resistance without drug induction. Redox Biol.

[66] Okoh V.O., Garba N.A., Penney R.B. (2015). Redox signalling to nuclear regulatory proteins by reactive oxygen species contributes to oestrogen-induced growth of breast cancer cells. Br J Cancer.

[67] Cao J.Y., Dixon S.J. (2016). Mechanisms of ferroptosis. Cell Mol Life Sci.

[68] De Blasio A., Di Fiore R., Pratelli G. (2020). A loop involving NRF2, miR-29b-1-5p and AKT, regulates cell fate of MDA-MB-231 triple-negative breast cancer cells. J Cell Physiol.

[69] Lee Y.S., Kang J., Jung E.S., Lee A. (2023). High expression of NRF2 and low expression of KEAP1 predict worse survival in patients with operable triple-negative breast cancer. J Breast Cancer.

[70] Qin S., He X., Lin H. (2021). Nrf2 inhibition sensitizes breast cancer stem cells to ionizing radiation via suppressing DNA repair. Free Radic Biol Med.

[71] Siraj M.A., Islam M.A., Al Fahad M.A., Kheya H.R., Xiao J., Simal-Gandara J. (2021). Cancer chemopreventive role of dietary terpenoids by modulating Keap1-Nrf2-ARE signaling system: a comprehensive update. Appl Sci.

[72] Peng F., Liao M., Qin R. (2022). Regulated cell death (RCD) in cancer: key pathways and targeted therapies. Signal Transduct Target Ther.

[73] Saddam M., Paul S.K., Habib M.A. (2024). Emerging biomarkers and potential therapeutics of the BCL-2 protein family: the apoptotic and anti-apoptotic context. Egypt J Med Hum Genet.

[74] Adinew G.M., Taka E., Mendonca P., Messeha S.S., Soliman K.F.A. (2021). The anticancer effects of flavonoids through miRNAs modulations in triple-negative breast cancer. Nutrients.

[75] García-Aranda M., Pérez-Ruiz E., Redondo M. (2018). Bcl-2 inhibition to overcome resistance to chemo- and immunotherapy. Int J Mol Sci.

[76] Safi A., Heidarian E., Ahmadi R. (2021). Quercetin synergistically enhances the anticancer efficacy of docetaxel through induction of apoptosis and modulation of PI3K/AKT, MAPK/ERK, and JAK/STAT3 signaling pathways in MDA-MB-231 breast cancer cell line. Int J Mol Cell Med.

[77] Kaloni D., Diepstraten S.T., Strasser A., Kelly G.L. (2023). BCL-2 protein family: attractive targets for cancer therapy. Apoptosis.

[78] Radha G., Raghavan S.C. (2017). BCL2: a promising cancer therapeutic target. Biochim Biophys Acta Rev Cancer.

[79] Khan M.A., Jain V.K., Rizwanullah M., Ahmad J., Jain K. (2019). PI3K/AKT/mTOR pathway inhibitors in triple-negative breast cancer: a review on drug discovery and future challenges. Drug Discov Today.

[80] Li H., Liu L., Chang H., Zou Z., Xing D. (2018). Downregulation of MCL-1 and upregulation of *PUMA* using mTOR inhibitors enhance antitumor efficacy of BH3 mimetics in triple-negative breast cancer. Cell Death Dis.

[81] Nakajima W., Miyazaki K., Sakaguchi M. (2022). Epigenetic priming with decitabine augments the therapeutic effect of cisplatin on triple-negative breast cancer cells through induction of proapoptotic factor NOXA. Cancers.

[82] Selmin O.I., Donovan M.G., Stillwater B.J., Neumayer L., Romagnolo D.F. (2020). Epigenetic regulation and dietary control of triple negative breast cancer. Front Nutr.

[83] Rivlin N., Brosh R., Oren M., Rotter V. (2011). Mutations in the p53 tumor suppressor gene: important milestones at the various steps of tumorigenesis. Genes Cancer.

[84] Beroukhim R., Mermel C.H., Porter D. (2010). The landscape of somatic copy-number alteration across human cancers. Nature.

[85] Campbell K.J., Tait S.W.G. (2018). Targeting BCL-2 regulated apoptosis in cancer. Open Biol.

[86] Zhang H., Xue J., Hessler P. (2015). Genomic analysis and selective small molecule inhibition identifies BCL-X(L) as a critical survival factor in a subset of colorectal cancer. Mol Cancer.

[87] Goodwin C.M., Rossanese O.W., Olejniczak E.T., Fesik S.W. (2015). Myeloid cell leukemia-1 is an important apoptotic survival factor in triple-negative breast cancer. Cell Death Differ.

[88] Lucantoni F., Lindner A.U., O’Donovan N., Düssmann H., Prehn J.H.M. (2018). Systems modeling accurately predicts responses to genotoxic agents and their synergism with BCL-2 inhibitors in triple negative breast cancer cells. Cell Death Dis.

[89] Bellaye P.S., Oudot A., Vrigneaud J.M. (2018). Nuclear imaging study of the pharmacodynamic effects of debio 1143, an antagonist of multiple inhibitor of apoptosis proteins (IAPs), in a triple-negative breast cancer model. Contrast Media Mol Imaging.

[90] Jo S.J., Park P.G., Cha H.R. (2017). Cellular inhibitor of apoptosis protein 2 promotes the epithelial-mesenchymal transition in triple-negative breast cancer cells through activation of the AKT signaling pathway. Oncotarget.

[91] Dumétier B., Zadoroznyj A., Dubrez L. (2020). IAP-mediated protein ubiquitination in regulating cell signaling. Cells.

[92] Fulda S. (2015). Targeting apoptosis for anticancer therapy. Semin Cancer Biol.

[93] Abbas R., Larisch S. (2021). Killing by degradation: regulation of apoptosis by the ubiquitin-proteasome-system. Cells.

[94] Kumar S., Fairmichael C., Longley D.B., Turkington R.C. (2020). The multiple roles of the IAP super-family in cancer. Pharmacol Ther.

[95] Allensworth J.L., Sauer S.J., Lyerly H.K., Morse M.A., Devi G.R. (2013). Smac mimetic Birinapant induces apoptosis and enhances TRAIL potency in inflammatory breast cancer cells in an IAP-dependent and TNF-α-independent mechanism. Breast Cancer Res Treat.

[96] Fulda S., Vucic D. (2012). Targeting IAP proteins for therapeutic intervention in cancer. Nat Rev Drug Discov.

[97] Xing J., Qi L., Liu X., Shi G., Sun X., Yang Y. (2022). Roles of mitochondrial fusion and fission in breast cancer progression: a systematic review. World J Surg Oncol.

[98] Deng Y., Ngo D.T.M., Holien J.K., Lees J.G., Lim S.Y. (2022). Mitochondrial dynamin-related protein Drp1: a new player in cardio-oncology. Curr Oncol Rep.

[99] Qian W., Wang J., Roginskaya V. (2014). Novel combination of mitochondrial division inhibitor 1 (mdivi-1) and platinum agents produces synergistic pro-apoptotic effect in drug resistant tumor cells. Oncotarget.

[100] Chiu H.Y., Tay E.X.Y., Ong D.S.T., Taneja R. (2020). Mitochondrial dysfunction at the center of cancer therapy. Antioxid Redox Signal.

[101] Zhang J., Liu Y., Tan J. (2021). Necroptotic virotherapy of oncolytic alphavirus M1 cooperated with doxorubicin displays promising therapeutic efficacy in TNBC. Oncogene.

[102] Romani P., Nirchio N., Arboit M. (2022). Mitochondrial fission links ECM mechanotransduction to metabolic redox homeostasis and metastatic chemotherapy resistance. Nat Cell Biol.

[103] Wu D., Dasgupta A., Chen K.H. (2020). Identification of novel dynamin-related protein 1 (Drp1) GTPase inhibitors: therapeutic potential of Drpitor1 and Drpitor1a in cancer and cardiac ischemia-reperfusion injury. FASEB J.

[104] Cassidy-Stone A., Chipuk J.E., Ingerman E. (2008). Chemical inhibition of the mitochondrial division dynamin reveals its role in Bax/Bak-dependent mitochondrial outer membrane permeabilization. Dev Cell.

[105] Rossi T., Iorio E., Chirico M. (2024). BET inhibitors (BETi) influence oxidative phosphorylation metabolism by affecting mitochondrial dynamics leading to alterations in apoptotic pathways in triple-negative breast cancer (TNBC) cells. Cell Prolif.

[106] Rocca C., De Francesco E.M., Pasqua T. (2022). Mitochondrial determinants of anti-cancer drug-induced cardiotoxicity. Biomedicines.

[107] Lucantoni F., Dussmann H., Prehn J.H.M. (2018). Metabolic targeting of breast cancer cells with the 2-deoxy-D-glucose and the mitochondrial bioenergetics inhibitor MDIVI-1. Front Cell Dev Biol.

[108] Yu S., Cao Z., Cai F. (2024). ADT-OH exhibits anti-metastatic activity on triple-negative breast cancer by combinatorial targeting of autophagy and mitochondrial fission. Cell Death Dis.

[109] Tang Q., Liu W., Zhang Q. (2018). Dynamin-related protein 1-mediated mitochondrial fission contributes to IR-783-induced apoptosis in human breast cancer cells. J Cell Mol Med.

[110] Shen L.W., Jiang X.X., Li Z.Q. (2022). Cepharanthine sensitizes human triple negative breast cancer cells to chemotherapeutic agent epirubicin via inducing cofilin oxidation-mediated mitochondrial fission and apoptosis. Acta Pharmacol Sin.

[111] Chernikova S.B., Nguyen R.B., Truong J.T. (2018). Dynamin impacts homology-directed repair and breast cancer response to chemotherapy. J Clin Invest.

[112] Si L., Liu W., Hayashi T. (2019). Silibinin-induced apoptosis of breast cancer cells involves mitochondrial impairment. Arch Biochem Biophys.

[113] Si L., Fu J., Liu W. (2020). Silibinin-induced mitochondria fission leads to mitophagy, which attenuates silibinin-induced apoptosis in MCF-7 and MDA-MB-231 cells. Arch Biochem Biophys.

[114] Si L., Fu J., Liu W. (2020). Silibinin inhibits migration and invasion of breast cancer MDA-MB-231 cells through induction of mitochondrial fusion. Mol Cell Biochem.

[115] Baek M.L., Lee J., Pendleton K.E. (2023). Mitochondrial structure and function adaptation in residual triple negative breast cancer cells surviving chemotherapy treatment. Oncogene.

[116] Humphries B.A., Cutter A.C., Buschhaus J.M. (2020). Enhanced mitochondrial fission suppresses signaling and metastasis in triple-negative breast cancer. Breast Cancer Res.

[117] Miret-Casals L., Sebastián D., Brea J. (2018). Identification of new activators of mitochondrial fusion reveals a link between mitochondrial morphology and pyrimidine metabolism. Cell Chem Biol.

[118] Park J.D., Jang H.J., Choi S.H. (2023). The ELK3-DRP1 axis determines the chemosensitivity of triple-negative breast cancer cells to CDDP by regulating mitochondrial dynamics. Cell Death Discov.

[119] Ding M., Feng N., Tang D. (2018). Melatonin prevents Drp1-mediated mitochondrial fission in diabetic hearts through SIRT1-PGC1α pathway. J Pineal Res.

[120] Pelicano H., Zhang W., Liu J. (2014). Mitochondrial dysfunction in some triple-negative breast cancer cell lines: role of mTOR pathway and therapeutic potential. Breast Cancer Res.

[121] Chelakkot C., Chelakkot V.S., Shin Y., Song K. (2023). Modulating glycolysis to improve cancer therapy. Int J Mol Sci.

[122] Li J., Eu J.Q., Kong L.R. (2020). Targeting metabolism in cancer cells and the tumour microenvironment for cancer therapy. Molecules.

[123] Fujita M., Imadome K., Somasundaram V., Kawanishi M., Karasawa K., Wink D.A. (2020). Metabolic characterization of aggressive breast cancer cells exhibiting invasive phenotype: impact of non-cytotoxic doses of 2-DG on diminishing invasiveness. BMC Cancer.

[124] O’Neill S., Porter R.K., McNamee N., Martinez V.G., O’Driscoll L. (2019). 2-Deoxy-D-Glucose inhibits aggressive triple-negative breast cancer cells by targeting glycolysis and the cancer stem cell phenotype. Sci Rep.

[125] Zhao Y., Butler E.B., Tan M. (2013). Targeting cellular metabolism to improve cancer therapeutics. Cell Death Dis.

[126] Yang Y. (2023). Targeting metabolic pathways improves TNBC therapy. Highlights Sci Eng Technol.

[127] Feng X., Zhang Y., Wang P., Liu Q., Wang X. (2014). Energy metabolism targeted drugs synergize with photodynamic therapy to potentiate breast cancer cell death. Photochem Photobiol Sci.

[128] Liu S., Li Y., Yuan M., Song Q., Liu M. (2023). Correlation between the Warburg effect and progression of triple-negative breast cancer. Front Oncol.

[129] Srivastava N., Usmani S.S., Subbarayan R., Saini R., Pandey P.K. (2023). Hypoxia: syndicating triple negative breast cancer against various therapeutic regimens. Front Oncol.

[130] Nedeljković M., Damjanović A. (2019). Mechanisms of chemotherapy resistance in triple-negative breast cancer-how we can rise to the challenge. Cells.

[131] Semenza G.L. (2017). Hypoxia-inducible factors: coupling glucose metabolism and redox regulation with induction of the breast cancer stem cell phenotype. EMBO J.

[132] Kaboli P.J., Imani S., Jomhori M., Ling K.H. (2021). Chemoresistance in breast cancer: PI3K/Akt pathway inhibitors *vs* the current chemotherapy. Am J Cancer Res.

[133] Guerrero-Zotano A., Mayer I.A., Arteaga C.L. (2016). PI3K/AKT/mTOR: role in breast cancer progression, drug resistance, and treatment. Cancer Metastasis Rev.

[134] Chen Q., Kirk K., Shurubor Y.I. (2018). Rewiring of glutamine metabolism is a bioenergetic adaptation of human cells with mitochondrial DNA mutations. Cell Metab.

[135] Gonsalves W.I., Jang J.S., Jessen E. (2020). *In vivo* assessment of glutamine anaplerosis into the TCA cycle in human pre-malignant and malignant clonal plasma cells. Cancer Metab.

[136] Zhou W.X., Chen C., Liu X.Q. (2021). Discovery and optimization of withangulatin A derivatives as novel glutaminase 1 inhibitors for the treatment of triple-negative breast cancer. Eur J Med Chem.

[137] Shah R., Chen S. (2020). Metabolic signaling cascades prompted by glutaminolysis in cancer. Cancers.

[138] Wang Z., Liu F., Fan N. (2020). Targeting glutaminolysis: new perspectives to understand cancer development and novel strategies for potential target therapies. Front Oncol.

[139] Dos Reis L.M., Adamoski D., Ornitz Oliveira Souza R. (2019). Dual inhibition of glutaminase and carnitine palmitoyltransferase decreases growth and migration of glutaminase inhibition-resistant triple-negative breast cancer cells. J Biol Chem.

[140] Singleton D.C., Dechaume A.L., Murray P.M., Katt W.P., Baguley B.C., Leung E.Y. (2020). Pyruvate anaplerosis is a mechanism of resistance to pharmacological glutaminase inhibition in triple-receptor negative breast cancer. BMC Cancer.

[141] Lampa M., Arlt H., He T. (2017). Glutaminase is essential for the growth of triple-negative breast cancer cells with a deregulated glutamine metabolism pathway and its suppression synergizes with mTOR inhibition. PLoS One.

[142] Hong J., Shen Y.A., Hsu C.Y. (2022). Targeting glutamine metabolism enhances responses to platinum-based chemotherapy in triple-negative breast cancers (TNBC). Genes Dis.

[143] Gross M.I., Demo S.D., Dennison J.B. (2014). Antitumor activity of the glutaminase inhibitor CB-839 in triple-negative breast cancer. Mol Cancer Therapeut.

[144] Jin J., Byun J.K., Choi Y.K., Park K.G. (2023). Targeting glutamine metabolism as a therapeutic strategy for cancer. Exp Mol Med.

[145] Kawakami I., Yoshino H., Fukumoto W. (2022). Targeting of the glutamine transporter SLC1A5 induces cellular senescence in clear cell renal cell carcinoma. Biochem Biophys Res Commun.

[146] Edwards D.N., Ngwa V.M., Raybuck A.L. (2021). Selective glutamine metabolism inhibition in tumor cells improves antitumor T lymphocyte activity in triple-negative breast cancer. J Clin Invest.

[147] Tang Y., Wang S., Li Y. (2022). Simultaneous glutamine metabolism and PD-L1 inhibition to enhance suppression of triple-negative breast cancer. J Nanobiotechnology.

[148] Halama A., Kulinski M., Dib S.S. (2018). Accelerated lipid catabolism and autophagy are cancer survival mechanisms under inhibited glutaminolysis. Cancer Lett.

[149] Nguyen T.T., Katt W.P., Cerione R.A. (2023). Alone and together: current approaches to targeting glutaminase enzymes as part of anti-cancer therapies. Future Drug Disc.

[150] Park S.Y., Choi J.H., Nam J.S. (2019). Targeting cancer stem cells in triple-negative breast cancer. Cancers.

[151] Sirois I., Aguilar-Mahecha A., Lafleur J. (2019). A unique morphological phenotype in chemoresistant triple-negative breast cancer reveals metabolic reprogramming and PLIN4 expression as a molecular vulnerability. Mol Cancer Res.

[152] Zhang Y., Wu M.J., Lu W.C., Li Y.C., Chang C.J., Yang J.Y. (2024). Metabolic switch regulates lineage plasticity and induces synthetic lethality in triple-negative breast cancer. Cell Metab.

[153] Loo S.Y., Toh L.P., Xie W.H. (2021). Fatty acid oxidation is a druggable gateway regulating cellular plasticity for driving metastasis in breast cancer. Sci Adv.

[154] Shin M.K., Cheong J.H. (2019). Mitochondria-centric bioenergetic characteristics in cancer stem-like cells. Arch Pharm Res.

[155] Butler E.B., Zhao Y., Muñoz-Pinedo C., Lu J., Tan M. (2013). Stalling the engine of resistance: targeting cancer metabolism to overcome therapeutic resistance. Cancer Res.

[156] Kéri G., Erchegyi J., Horváth A. (1996). A tumor-selective somatostatin analog (TT-232) with strong *in vitro* and *in vivo* antitumor activity. Proc Natl Acad Sci U S A.

[157] Chen J., Xie J., Jiang Z., Wang B., Wang Y., Hu X. (2011). Shikonin and its analogs inhibit cancer cell glycolysis by targeting tumor pyruvate kinase-M2. Oncogene.

[158] Guan M., Tong Y., Guan M. (2018). Lapatinib inhibits breast cancer cell proliferation by influencing PKM2 expression. Technol Cancer Res Treat.

[159] Ahmad Siddiqui F., Prakasam G., Chattopadhyay S. (2018). Curcumin decreases Warburg effect in cancer cells by down-regulating pyruvate kinase M2 via mTOR-HIF1α inhibition. Sci Rep.

[160] Tan J., Li Z., Lee P.L. (2013). PDK1 signaling toward PLK1-MYC activation confers oncogenic transformation, tumor-initiating cell activation, and resistance to mTOR-targeted therapy. Cancer Discov.

[161] Sun R.C., Fadia M., Dahlstrom J.E., Parish C.R., Board P.G., Blackburn A.C. (2010). Reversal of the glycolytic phenotype by dichloroacetate inhibits metastatic breast cancer cell growth *in vitro* and *in vivo*. Breast Cancer Res Treat.

[162] Umar S.M., Kashyap A., Kahol S. (2020). Prognostic and therapeutic relevance of phosphofructokinase platelet-type (PFKP) in breast cancer. Exp Cell Res.

[163] Coelho R.G., de Castro Calaça I., de Moura Celestrini D., Correia A.H., Costa M.A.S.M., Sola-Penna M. (2011). Clotrimazole disrupts glycolysis in human breast cancer without affecting non-tumoral tissues. Mol Genet Metabol.

[164] Furtado C.M., Marcondes M.C., Sola-Penna M., de Souza M.L.S., Zancan P. (2012). Clotrimazole preferentially inhibits human breast cancer cell proliferation, viability and glycolysis. PLoS One.

[165] Meira D.D., Marinho-Carvalho M.M., Teixeira C.A. (2005). Clotrimazole decreases human breast cancer cells viability through alterations in cytoskeleton-associated glycolytic enzymes. Mol Genet Metabol.

[166] Spitz G.A., Furtado C.M., Sola-Penna M., Zancan P. (2009). Acetylsalicylic acid and salicylic acid decrease tumor cell viability and glucose metabolism modulating 6-phosphofructo-1-kinase structure and activity. Biochem Pharmacol.

[167] Xintaropoulou C., Ward C., Wise A., Marston H., Turnbull A., Langdon S.P. (2015). A comparative analysis of inhibitors of the glycolysis pathway in breast and ovarian cancer cell line models. Oncotarget.

[168] Falcone I.G., Rushing B.R. (2025). Untargeted metabolomics reveals acylcarnitines as major metabolic targets of resveratrol in breast cancer cells. Metabolites.

[169] Kwiatkowska E., Wojtala M., Gajewska A., Soszyński M., Bartosz G., Sadowska-Bartosz I. (2016). Effect of 3-bromopyruvate acid on the redox equilibrium in non-invasive MCF-7 and invasive MDA-MB-231 breast cancer cells. J Bioenerg Biomembr.

[170] Yousefi S., Darvishi P., Yousefi Z., Pourfathollah A.A. (2020). Effect of methyl jasmonate and 3-bromopyruvate combination therapy on mice bearing the 4 T1 breast cancer cell line. J Bioenerg Biomembr.

[171] Li W., Zheng M., Wu S. (2017). Benserazide, a dopadecarboxylase inhibitor, suppresses tumor growth by targeting hexokinase 2. J Exp Clin Cancer Res.

[172] Koh M., Lee J.C., Min C., Moon A. (2013). A novel metformin derivative, HL010183, inhibits proliferation and invasion of triple-negative breast cancer cells. Bioorg Med Chem.

[173] Marini C., Salani B., Massollo M. (2013). Direct inhibition of hexokinase activity by metformin at least partially impairs glucose metabolism and tumor growth in experimental breast cancer. Cell Cycle.

[174] Wahdan-Alaswad R.S., Edgerton S.M., Salem H.S., Thor A.D. (2018). Metformin targets glucose metabolism in triple negative breast cancer. J Oncol Transl Res.

[175] Deng X.S., Wang S., Deng A. (2012). Metformin targets Stat3 to inhibit cell growth and induce apoptosis in triple-negative breast cancers. Cell Cycle.

[176] Silvestri A., Palumbo F., Rasi I. (2015). Metformin induces apoptosis and downregulates pyruvate kinase M2 in breast cancer cells only when grown in nutrient-poor conditions. PLoS One.

[177] Varghese E., Samuel S.M., Líšková A., Samec M., Kubatka P., Büsselberg D. (2020). Targeting glucose metabolism to overcome resistance to anticancer chemotherapy in breast cancer. Cancers.

[178] Nath K., Nelson D.S., Heitjan D.F., Leeper D.B., Zhou R., Glickson J.D. (2015). Lonidamine induces intracellular tumor acidification and ATP depletion in breast, prostate and ovarian cancer xenografts and potentiates response to doxorubicin. NMR Biomed.

[179] Cohen-Erez I., Issacson C., Lavi Y. (2019). Antitumor effect of lonidamine-polypeptide-peptide nanoparticles in breast cancer models. ACS Appl Mater Interfaces.

[180] Christenson J.L., Butterfield K.T., Spoelstra N.S. (2017). MMTV-PyMT and derived met-1 mouse mammary tumor cells as models for studying the role of the androgen receptor in triple-negative breast cancer progression. Horm Cancer.

[181] Molina J.R., Sun Y., Protopopova M. (2018). An inhibitor of oxidative phosphorylation exploits cancer vulnerability. Nat Med.

[182] Gao W., Zhang J., Wang W. (2022). Drug self-delivery nanorods enhance photodynamic therapy of triple-negative breast cancer by inhibiting oxidative phosphorylation. Int J Pharm.

[183] Louie S.M., Grossman E.A., Crawford L.A. (2016). GSTP1 is a driver of triple-negative breast cancer cell metabolism and pathogenicity. Cell Chem Biol.

[184] Thornburg J.M., Nelson K.K., Clem B.F. (2008). Targeting aspartate aminotransferase in breast cancer. Breast Cancer Res.

[185] Zhou M., Zhao Y., Ding Y. (2010). Warburg effect in chemosensitivity: targeting lactate dehydrogenase-A re-sensitizes Taxol-resistant cancer cells to Taxol. Mol Cancer.

[186] Farabegoli F., Vettraino M., Manerba M., Fiume L., Roberti M., Di Stefano G. (2012). Galloflavin, a new lactate dehydrogenase inhibitor, induces the death of human breast cancer cells with different glycolytic attitude by affecting distinct signaling pathways. Eur J Pharmaceut Sci.

[187] Giri P., Camarillo I.G., Mitta L., Sundararajan R. (2022). Quantitative proteomic assessment of key proteins regulated by electrical pulse-mediated galloflavin delivery in triple-negative breast cancer cells. Biointerface Res Appl Chem.

[188] Olayanju A., Copple I.M., Bryan H.K. (2015). Brusatol provokes a rapid and transient inhibition of Nrf2 signaling and sensitizes mammalian cells to chemical toxicity-implications for therapeutic targeting of Nrf2. Free Radic Biol Med.

[189] Xie J., Lai Z., Zheng X. (2021). Apoptotic activities of brusatol in human non-small cell lung cancer cells: involvement of ROS-mediated mitochondrial-dependent pathway and inhibition of Nrf2-mediated antioxidant response. Toxicology.

[190] Li J., Zhang J., Zhu Y., Afolabi L.O., Chen L., Feng X. (2023). Natural compounds, optimal combination of brusatol and polydatin promote anti-tumor effect in breast cancer by targeting Nrf2 signaling pathway. Int J Mol Sci.

[191] Chandrasekaran J., Balasubramaniam J., Sellamuthu A., Ravi A. (2021). An *in vitro* study on the reversal of epithelial to mesenchymal transition by brusatol and its synergistic properties in triple-negative breast cancer cells. J Pharm Pharmacol.

[192] Waghela B.N., Vaidya F.U., Pathak C. (2021). Upregulation of NOX-2 and Nrf-2 promotes 5-fluorouracil resistance of human colon carcinoma (HCT-116) cells. Biochemistry.

[193] Woo Y., Lee H.J., Jung Y.M., Jung Y.J. (2019). mTOR-mediated antioxidant activation in solid tumor radioresistance. J Oncol.

[194] Wakimoto R., Ono M., Takeshima M., Higuchi T., Nakano S. (2017). Differential anticancer activity of pterostilbene against three subtypes of human breast cancer cells. Anticancer Res.

[195] Hung C.M., Liu L.C., Ho C.T., Lin Y.C., Way T.D. (2017). Pterostilbene enhances TRAIL-induced apoptosis through the induction of death receptors and downregulation of cell survival proteins in TRAIL-resistance triple negative breast cancer cells. J Agric Food Chem.

[196] Foygel K., Sekar T.V., Paulmurugan R. (2015). Monitoring the antioxidant mediated chemosensitization and ARE-signaling in triple negative breast cancer therapy. PLoS One.

[197] Kwon Y. (2021). Possible beneficial effects of N-acetylcysteine for treatment of triple-negative breast cancer. Antioxidants.

[198] Hata A.N., Engelman J.A., Faber A.C. (2015). The BCL2 family: key mediators of the apoptotic response to targeted anticancer therapeutics. Cancer Discov.

[199] Henz K., Al-Zebeeby A., Basoglu M. (2019). Selective BH3-mimetics targeting BCL-2, BCL-XL or MCL-1 induce severe mitochondrial perturbations. Biol Chem.

[200] Cerella C., Dicato M., Diederich M. (2020). BH3 mimetics in AML therapy: death and beyond?. Trends Pharmacol Sci.

[201] Al-Zebeeby A., Vogler M., Milani M. (2019). Targeting intermediary metabolism enhances the efficacy of BH3 mimetic therapy in hematologic malignancies. Haematologica.

[202] Hiraki M., Suzuki Y., Alam M. (2016). MUC1-C stabilizes MCL-1 in the oxidative stress response of triple-negative breast cancer cells to BCL-2 inhibitors. Sci Rep.

[203] Inao T., Iida Y., Moritani T. (2018). Bcl-2 inhibition sensitizes triple-negative human breast cancer cells to doxorubicin. Oncotarget.

[204] Karpel-Massler G., Ishida C.T., Bianchetti E. (2017). Inhibition of mitochondrial matrix chaperones and antiapoptotic Bcl-2 family proteins empower antitumor therapeutic responses. Cancer Res.

[205] Lee A., Jin H.O., Masudul Haque M. (2022). Synergism of a novel MCL-1 downregulator, acriflavine, with navitoclax (ABT-263) in triple-negative breast cancer, lung adenocarcinoma and glioblastoma multiforme. Int J Oncol.

[206] Wali V.B., Langdon C.G., Held M.A. (2017). Systematic drug screening identifies tractable targeted combination therapies in triple-negative breast cancer. Cancer Res.

[207] Hamunyela R.H., Serafin A.M., Akudugu J.M. (2017). Strong synergism between small molecule inhibitors of HER2, PI3K, mTOR and Bcl-2 in human breast cancer cells. Toxicol In Vitro.

[208] Xie X., Lee J., Liu H. (2021). Birinapant enhances gemcitabine’s antitumor efficacy in triple-negative breast cancer by inducing intrinsic pathway-dependent apoptosis. Mol Cancer Therapeut.

[209] Tao Z., McCall N.S., Wiedemann N., Vuagniaux G., Yuan Z., Lu B. (2019). SMAC mimetic debio 1143 and ablative radiation therapy synergize to enhance antitumor immunity against lung cancer. Clin Cancer Res.

[210] Wang S., Bai L., Lu J., Liu L., Yang C.Y., Sun H. (2012). Targeting inhibitors of apoptosis proteins (IAPs) for new breast cancer therapeutics. J Mammary Gland Biol Neoplasia.

[211] Cai Q., Sun H., Peng Y. (2011). A potent and orally active antagonist (SM-406/AT-406) of multiple inhibitor of apoptosis proteins (IAPs) in clinical development for cancer treatment. J Med Chem.

